# Aberrant Scinderin Expression Correlates With Liver Metastasis and Poor Prognosis in Colorectal Cancer

**DOI:** 10.3389/fphar.2019.01183

**Published:** 2019-10-31

**Authors:** Qi Lin, Jun Li, Dexiang Zhu, Zhengchuan Niu, Xiangou Pan, Pingping Xu, Meiling Ji, Ye Wei, Jianmin Xu

**Affiliations:** ^1^Department of General Surgery, Zhongshan Hospital, Fudan University, Shanghai, China; ^2^Department of Radiology, Zhongshan Hospital, Fudan University, Shanghai, China

**Keywords:** scinderin, colorectal cancer, liver metastasis, genome-wide association study, prognosis

## Abstract

Many genes and mutations have been reported for colorectal cancer (CRC); however, very few have been associated with colorectal cancer liver metastasis (CRLM). We performed gene expression profiling experiments to identify genetic markers for CRLM and elucidate the molecular mechanisms. Microarray experiments were performed on CRC primary tumor samples with or without liver metastasis (LM) using the Affymetrix U133 plus 2.0 GeneChip Array. A new identified gene-scinderin (*SCIN*) was overexpressed with synchronous LM at both the RNA level evaluated with quantitative real-time PCR and protein level evaluated with immunohistochemistry and also with short overall survival analyzed with Kaplan-Meier method. With multivariate analysis indicated that *SCIN* served as an independent poor prognostic predictor for CRC patients. Disease-free survival was also significantly lower in *SCIN* overexpressing CRC patients with metachronous LM. In addition, *SCIN* knockdown significantly reduced cell proliferation, induced cell cycle arrest, and promoted the expression of some cell cycle apoptosis-related protein. Moreover, the *DIAPH1*, *STAT3*, *CDK2*, *CDK4*, and *EGFR* levels were downregulated, whereas *CDKN2B* and *COL4A1* were upregulated in DLD-1-shSCIN cells by microarray analysis compared with DLD-1 shCon cells. These findings revealed that *SCIN* may serve as an important predictor of CRLM and poor outcome for CRC patients. *SCIN* may be a potential therapeutic target in human CRC. However, translation of its roles into clinical practice will require further investigation and additional experimental validation.

## Introduction

Even in developed countries, colorectal cancer (CRC) is the third most common malignancy diagnosed in both men and women and is the second leading cause of cancer deaths (Siegel et al., 2013). Approximately 25% of patients with CRC present with liver metastasis (LM) at initial diagnosis and almost 50% will develop LM. Approximately, 25%–50% of patients with surgically resected colorectal cancer liver metastasis (CRLM) survive 5 or more years (Manfredi et al., 2006; Robertson et al., 2009). Even after radical resection, relapse can occur in 75% of patients, generally occurring within the first 2 years after surgery, and 50% of relapses are in the liver (Petrelli, 2008). Therefore, identifying biomarkers for early CRLM diagnosis and those with a prognostic value for CRC after resection may help patients choose a suitable follow-up interval and subsequent therapy.

Scinderin (*SCIN*), or adseverin, was initially identified as a calcium-regulated, actin depolymerizing agent involved in secretion; it was named in response to its original isolation from the adrenal medulla. *SCIN* possesses the ability to sever actin filaments and is a member of the gelsolin family of actin-binding proteins (Maekawa and Sakai, 1990; Rodriguez Del Castillo et al., 1990); In addition, it can bind actin monomers and is present in secretory cells (Rodriguez Del Castillo et al., 1990). *SCIN* comprises six homologous domains (A1–A6) that share 60% identity to the six domains from gelsolin (G1–G6), which can inhibit mitochondrial apoptosis by closing voltage-dependent anion channels (VDACs) (Kusano et al., 2000). Miura et al. (2012) reported the interaction between *SCIN* and VDAC, particularly in acisplatin-resistant human bladder cancer cell line overexpressing *SCIN*, and this binding was suggested to contribute to cisplatin resistance *via* the inhibition of mitochondria-mediated apoptosis.

Recently, studies found that *SCIN* functioning in the development and progression of some human cancers. *SCIN* was highly expressed in gastric cancer tissues and the level of expression associated with the depth of tumor invasion, lymph node metastasis, and poor overall survival. *SCIN* knockdown inhibited the invasion and metastasis of gastric cancer cells and restrained the filopodium formation and Cdc42 expression (Liu et al., 2016). In hepatocellular carcinoma, *SCIN* knockdown sensitized cancer cells to chemotherapy and inhibited tumor growth *in vivo*. Consistently, *SCIN* overexpression protected cells from apoptosis, promoted xenografted tumor cell growth (Qiao et al., 2018). In prostate cancer, *SCIN* knockdown significantly downregulated the protein expression of epidermal growth factor receptor (EGFR), impaired cell proliferation-mediated by epidermal growth factor, and inhibited the signaling pathway activation of the downstream mitogen-activated protein kinase (MEK) and extracellular signal-regulated kinase (ERK). *SCIN* knockdown promoted prostate cell apoptosis by inhibition of B-cell lymphoma-extra-large (Bcl-xl) expression and caspase signaling (Lai et al., 2018). However, the clinical significance and molecular mechanism for *SCIN* in CRC remain unknown.

Based on the whole-genome expression profiling of CRC, we observed that *SCIN*, a newly recognized biomarker, was significantly upregulated in CRC patients with synchronous liver metastasis (SLM) and a poor prognosis. Thus, we further evaluated the potential molecular mechanisms responsible for these effects of *SCIN* in CRC cells.

## Materials and Methods

### Selection of Patient Material

Tumor specimens with patient clinical and follow-up data were selected from our tumor bank. We divided the samples into two groups according to the presence of LM. The samples were obtained from patients with no family history of CRC or secondary malignancy, and these patients had not received radiation or chemotherapy before surgery. The patients in the nonmetastatic group had a minimum of 3 years disease-free survival (DFS) after surgery. For additional details related to patient materials, see **Table 1** and **Supplementary Tables 1** and **3**.

**Table 1:** Clinicopathologic variables and *SCIN* expression in 300 CRCs.

Variables	*SCIN* expression		*P*-value
	High	Low	
Age (years)			
=60	172	32	0.507
>60	78	18	
Gender			
Male	119	23	0.836
Female	131	27	
Tumor location			
Colon	160	28	0.286
Rectum	90	22	
Tumor size			
=5 cm	184	37	0.953
>5 cm	66	13	
Gross appearance			
Ulcerative	190	37	0.764
Exophytic	60	13	
Histological type			
Adenocarcinoma	220	43	0.695
Mucinous adenocarcinoma	30	7	
Tumor differentiation			
Well or moderate	80	18	0.582
Poor or other	170	32	
Depth of invasion			
T1,2	13	36	<0.001
T3,4	237	14	
Lymph node metastasis			
Absent	107	30	0.026
Present	143	20	
CRC and LM			
CRC without LM	78	22	0.021^a^
CRC with SLM	90	10	0.103^b^
CRC with MLM	82	18	0.480^c^

a, CRC without LM vs. CRC with SLM P = 0.021.

b, CRC with SLM vs. CRC with MLM P = 0.480.

c, CRC with MLM vs. CRC without LM P = 0.103.

### Microarray Sample Preparation and Gene Expression Analysis

RNA isolation and microarray procedures were performed according to the manufacturer’s instructions, as previously described (Watanabe et al., 2006). Briefly, for sample preparation, snap-frozen tumor samples were cryosectioned (5 µm), and tumor tissues (~1 mm^2^) were collected using laser-capture microdissection. RNA was isolated from the microdissected material using Trizol (California Carlsbad, Invitrogen, USA) extraction, following the manufacturer’s guidelines. Microarray profiling was performed using human U133 Plus 2.0 GeneChip^(r)^ (Affymetrix, Santa Clara, CA) arrays according to the manufacturer’s protocol.

### Cell Lines, Plasmid Construction, and Transfection

The cell lines were obtained from the Chinese Academy of Sciences and cultured in 1640 medium with 10% fetal bovine serum (Logan Utah, HyClone, USA). The pGC-LV/GFP expression vector was purchased from Shanghai Genechem Ltd. *SCIN* cDNA was obtained from the RZPD clone bank (Germany). The interfering oligonucleotide designed with a short hairpin structure targeting *SCIN* and a scrambled shRNA as a control were cloned into the pGC-LV/GFP vector. The recombinant vector pGC-LV/GFP/-shRNA *SCIN* was confirmed by DNA sequencing and enzyme digestion analysis. DLD-1 and SW480 cells were transfected with pGC-LV/GFP/-shRNA *SCIN* and pGC-LV/GFP/-control using Lipofectamine2000 (Invitrogen), as recommended by the manufacturer.

### Quantitative Real-Time PCR

For quantitative real-time PCR (qRT-PCR) to measure the levels of *SCIN* RNA (Fritzmann et al., 2009), tumor cryosections of 60 CRC samples, including those without LM (n = 20), with SLM (n = 20), and with metachronous LM (MLM) (n = 20), were processed, and RNA was examined as previously described (Fritzmann et al., 2009). Gene-specific primers for PCR products were designed using PPRIMER5 software with information from GenBank (NCBI). The primer sequences are shown in **Supplementary Table 7**.

### Immunohistochemistry and Staining Evaluation

For *SCIN* protein expression analysis, formalin-fixed paraffin-embedded tissues from 300 patients (18–75 years old) including paired normal mucosa were used for immunohistochemistry (IHC) as previously described (Spano et al., 2005) (rabbit polyclonal anti-*SCIN*; Sigma Corporation, Cat#: HPA020518, USA).

The intensity of the IHC staining of *SCIN* was scored using the semiquantitative immunoreactivity scoring (IRS) system (Weichert et al., 2008). The percentage of labeled cells was graded as follows: grade 0, no positive cells; grade 1, 1%~25% labeled tumor cells; grade 2, 26%~50% labeled tumor cells; grade 3, 51%~75% labeled tumor cells; and grade 4, >75% positive tumor cells. The intensity of peroxidase deposits, ranging from light beige to dark brown, was assessed visually in the tumor cell cytoplasm and scored as 0 (negative), 1 (weak), 2 (moderate), or 3 (strong). A composite score, potentially ranging from 0 to 12, was obtained by multiplying the grade by the intensity. An IRS value between 0 and 6 was considered low expression, and IRS value >6 was considered high expression. All specimens were pathologically reassessed independently by two gastroenterology pathologists blinded to the clinical data.

### MTT Assay

The proliferation of transfected cells was measured using an MTT assay. A total of 10 µl of MTT (5 mg/ml; Sigma) was added to each well for a final volume of 100 µl of culture medium containing viable cells. After an additional incubation of 4 h, the resulting formazan was dissolved in 100 µl of isopropanol with 40 mM hydrochloric acid. Spectrophotometric absorbance at 570 nm (for formazan dye) was measured with absorbance at 630 nm as a reference.

### Western Blotting

Cells were harvested and scraped in RIPA buffer containing 10% protease inhibitor cocktail to obtain the total protein content. The BCA method was used to measure the protein concentration. Each sample was fractionated using 10% SDS-PAGE and blotted onto PVDF membranes. The membranes were incubated in 5% nonfat dry milk to block nonspecific binding and then blotted with a primary antibody overnight at 4°C. After washes with TBST and incubation with anti-rabbit horseradish peroxidase-conjugated secondary antibody (Biosynthesis Biotechnology, China) for 2 h at room temperature, the immunocomplexes were visualized using chemiluminescence (GE, USA) according to the manufacturer’s protocol (Sun et al., 2008).

### Flow Cytometry Analysis

Cell cycle progression was analyzed by flow cytometry. In brief, 4 days after lentivirus infection, cells were collected, washed by PBS, and fixed by 75% ethanol. Cells were stained with propidium iodide (PI) and RNase overnight at 4°C. Samples were then analyzed.

### Gene Expression Analysis Between DLD-1-Shscin and DLD-1-Shcon Cells

Total RNA was isolated from DLD-1-shSCIN and DLD-1-shCon cells. The RNA samples were converted to biotinylatedc RNA and hybridized to the GeneChip, as described above. Then, gene cluster analysis, gene ontology (GO) analysis (including molecular function, biological process and cellular component), and pathway analysis were applied between the differently expressed genes, and the discrepancy in gene expression was confirmed using qRT-PCR, as described above (Fritzmann et al., 2009).

### The Significance Analysis of Microarrays

The popular statistical technique of significance analysis of microarrays (SAM) applying to detect the differentially expressed genes was first introduced by Tusher et al. in 2001 (Tusher et al., 2001). SAM identifies gene by assimilating a set of gene-specific t-tests. Each gene is allocated a score on account of its change in gene expression associated to the standard deviation of repeated detection for that gene. Gene with score greater than an assumed threshold is considered potentially significant. The percentage of the gene identified by probability is the false discovery rate (FDR). The different number of genes can be identified by adjusting the threshold, and FDRs can be calculated for each set (Tusher et al., 2001; Rieger and Chu, 2004; Del Rio et al., 2007; Cescon et al., 2015; Steinberg et al., 2017; Feng et al., 2018; Hao et al., 2019).

In this study, a set of genes with a FDR < 0.05 were then selected and an absolute value (fold-change cutoff) of 2.7 was applied to reduce the number of potential probes between CRC patients with and without LM. Statistical analyses were performed using the open source software R version 2.13.1.

### Statistical Analyses

Continuous data were measured using a t-test. For categorical data, chi-squared analysis or Fisher’s exact test was used. The survival rate was analyzed with the Kaplan-Meier method, and differences in survival rates were assessed with the log-rank test. A Cox proportional hazards model was used for multivariate analysis. All statistical analyses were performed using SPSS 16.0 software (SPSS, Chicago, IL, USA). Two-sided *P*-values were calculated, and *P* < 0.05 was considered significant.

## Results

### *SCIN* Is Significantly Upregulated in SLM

**Figure 1:**
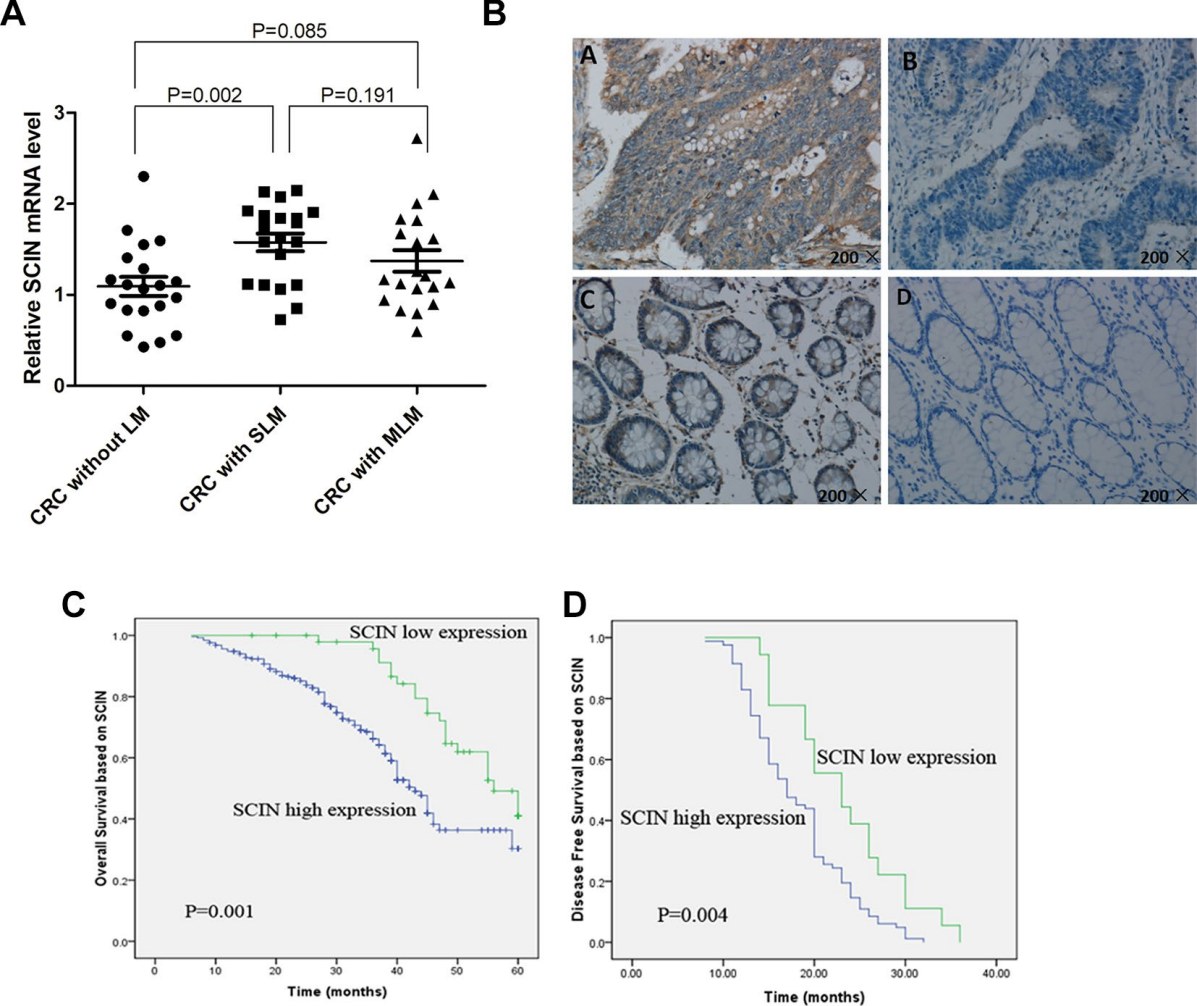
*SCIN* expression in CRC tissue samples and Kaplan-Meier analysis with OS and DFS. **(A)** qRT-PCR was used to detect *SCIN* mRNA expression levels in CRC primary tumor tissues without LM, with SLM, or with MLM. **(B)** Representative immunostains of *SCIN* expression in CRC clinical samples. Positive cells are stained brown high-intensity *SCIN* expression in cancerous tissues (upper left). Low-intensity *SCIN* expression in cancerous tissues (upper right). Positive *SCIN* staining in paracancerous tissue (below left). Negative *SCIN* staining in paracancerous tissue (below right). **(C)** Kaplan-Meier analysis of OS related to *SCIN* expression in patients with CRC (n = 300; *P* = 0.001). **(D)** DFS (*P* = 0.004) related to *SCIN* expression in MLM patients (n = 100).

Microarray profiling was performed using human U133 Plus 2.0 GeneChip^(r)^ in primary tumors of CRLM patients (n = 5) and CRC patients without LM (n = 6). We applied the SAM to analyze gene expression differences (for scores with an absolute value ≥2.7) and identified a list of 110 genes differentially expressed at significant levels in patients with and without LM (with different absolute value, different numbers of genes can be obtained; the baseline data of the GeneChip patients and differentially 110 genes were in **Supplementary Tables 1** and **2**, respectively). Of these, 60 genes (including the *SCIN*) were shown to have higher expression levels in patients with LM, and 50 genes were shown to have lower expression levels. Based on literature search, bioinformatics analysis, and patent database of cancer key gene, after excluding some Star Genes that have been studied, we focusing on 24 genes (*SCIN* is one of them). Then, we examined *SCIN* using a qRT-PCR method based on SYBR green for CRC primary tumors without LM (n = 20), with SLM (n = 20), and with MLM (n = 20) (The baseline data of the 60 patients examined by qRT-PCR was in **Supplementary Table 3**). We found that the *SCIN* was expressed at significantly higher level in CRC tumor samples with SLM compared to patients without LM (*P* = 0.002), no significantly different was found between patients without LM and MLM (*P* = 0.085), and no significantly different was found between SLM and MLM patients (*P* = 0.191) (**Figure 1A**). Then, we used IHC to examine *SCIN* protein expression levels in 300 CRC patients, including those without LM (n = 100), with SLM (n = 100), and with MLM (n = 100). We observed brown *SCIN* staining in the cancer cell cytoplasm, and very few paracancerous samples showed low expression levels (**Figure 1B**). In addition, we found that high *SCIN* expression levels in cancerous tissues were significantly associated with SLM compared to CRC patients without LM (*P* = 0.021) (**Table 1**).

### The Correlation Between *SCIN* Expression and Clinicopathologic Parameters

According to the extent of *SCIN* staining in the cytoplasm, the experimental samples were divided into two groups [the high *SCIN* expression group (n = 250) and the low *SCIN* expression group (n = 50)] to investigate *SCIN* expression in association with clinicopathologic variables. We found high *SCIN* expression was related to the depth of invasion and lymph node metastasis (*P* < 0.05), but was not related to age, gender, tumor location, tumor size, gross appearance, histological type, and tumor differentiation (*P* > 0.05; **Table 1**).

### *SCIN* Expression and Prognosis of CRC Patients

We applied Kaplan-Meier survival analysis to study the relationship between *SCIN* expression and overall survival (OS) among the 300 patients previously described. OS was significantly lower among *SCIN*-overexpressing patients (*P* = 0.001; **Figure 1C**). In addition to *SCIN* expression, univariate analysis also identified poor tumor differentiation, depth of invasion infiltration (T3-4), lymph node metastasis, and distant metastasis (SLM and MLM) as significantly associated with poor OS (**Table 2**). Other clinicopathologic features such as age, gender, tumor location, tumor size, gross appearance, and histological type were not significant prognostic factors (**Table 2**). **Table 2** also shows the results of multivariate analysis in the final model through the use of a multivariate Cox proportional hazard regression model, which included *SCIN* expression, tumor differentiation, depth of invasion infiltration, lymph node metastasis, and distant metastasis. In this model, the variables of high *SCIN* expression and distant metastasis were independent prognostic predictors for CRC patients. We also analyzed DFS among MLM patients using Kaplan-Meier survival analysis and found that DFS was significantly lower in patients with *SCIN* overexpression (*P* = 0.004; **Figure 1D**).

**Table 2:** Univariate and multivariate analysis of clinicopathological factors for OS in 300 CRC patients.

Characteristics	OS				
	Univariate analysis		Multivariate analysis		
	χ^2^	P	Exp(B)	95%CI for Exp(B)	P
Age	0.289	0.591			
Gender	3.632	0.057			
Tumor location	0.569	0.450			
Tumor size	0.946	0.331			
Gross appearance	0.036	0.849			
Histological type	0.300	0.584			
Tumor differentiation	5.620	0.018	0.918	0.590–1.428	0.704
Depth of invasion	4.745	0.029	0.826	0.457–1.492	0.526
Lymph node metastasis	11.118	0.001	0.715	0.475–1.077	0.109
With distant metastasis	79.905	<0.001	8.511	5.032–14.394	<0.001
*SCIN* expression	11.097	0.001	0.343	0.185–0.635	0.001

### *SCIN* Knockdown Inhibits Cell Proliferation and Induces Cell Cycle Arrest as Well as Cell Apoptosis

**Figure 2:**
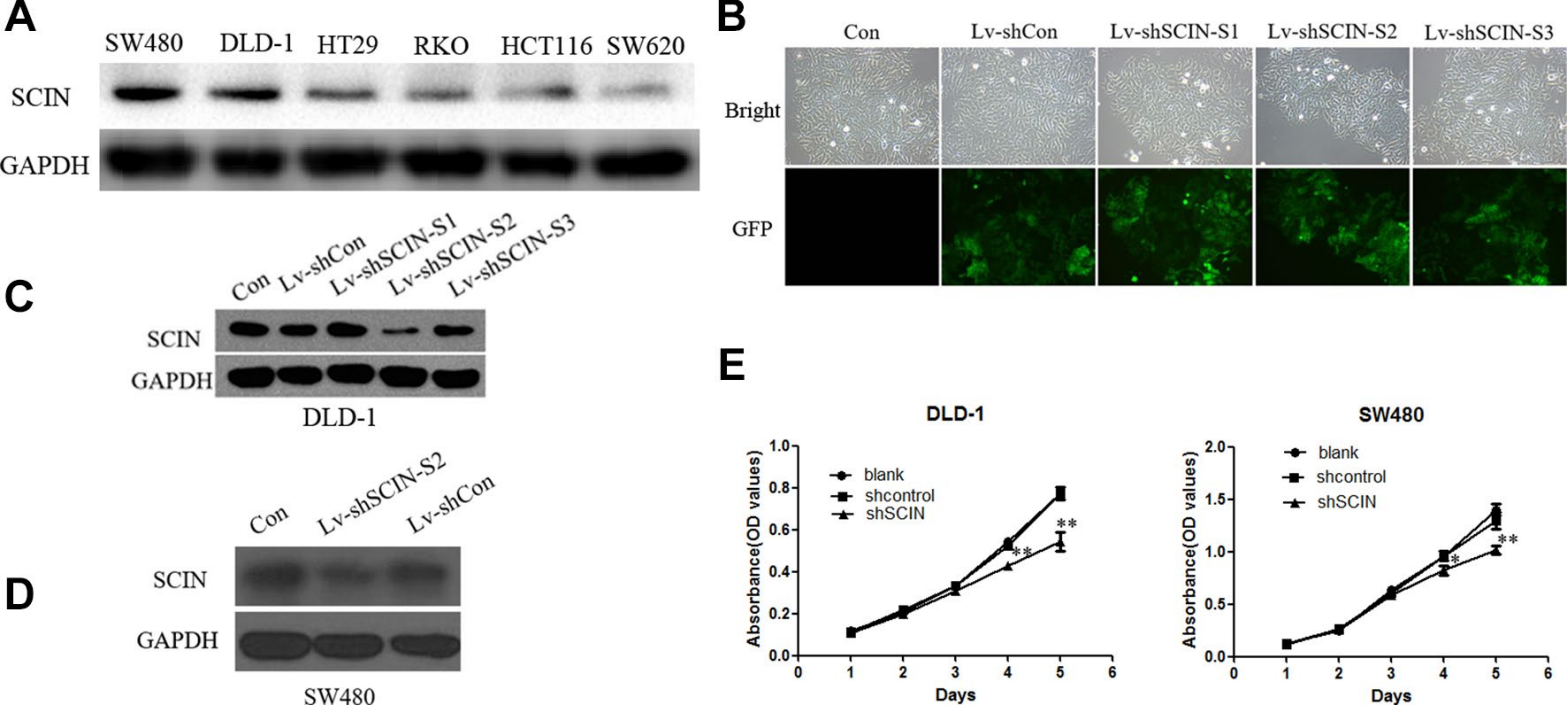
Depletion of SCIN in CRC cells by Lv-shSCIN infection. **(A)** Expression of *SCIN* in CRC cell lines (SW480, DLD-1, HT29, RKO, HCT116, and SW620), as revealed using Western blotting. **(B)** *SCIN* expression levels in DLD-1 cells transduced with Lv-shSCIN. Infection efficiencies were evaluated by GFP fluorescence in DLD-1 cells after lenti-virus infection. **(C)** The expression of *SCIN* was significantly inhibited after Lv-shSCIN-S2 was successfully transfected into DLD-1 cell line. **(D)** The expression of *SCIN* was significantly inhibited after Lv-shSCIN-S2 was successfully transfected into SW480 cell line. **(E)** *SCIN* knockdown significantly reduced cell proliferation in the cell line of DLD-1 and SW480, as determined using an MTT assay. (*P < 0.05, **P < 0.01).

**Figure 3:**
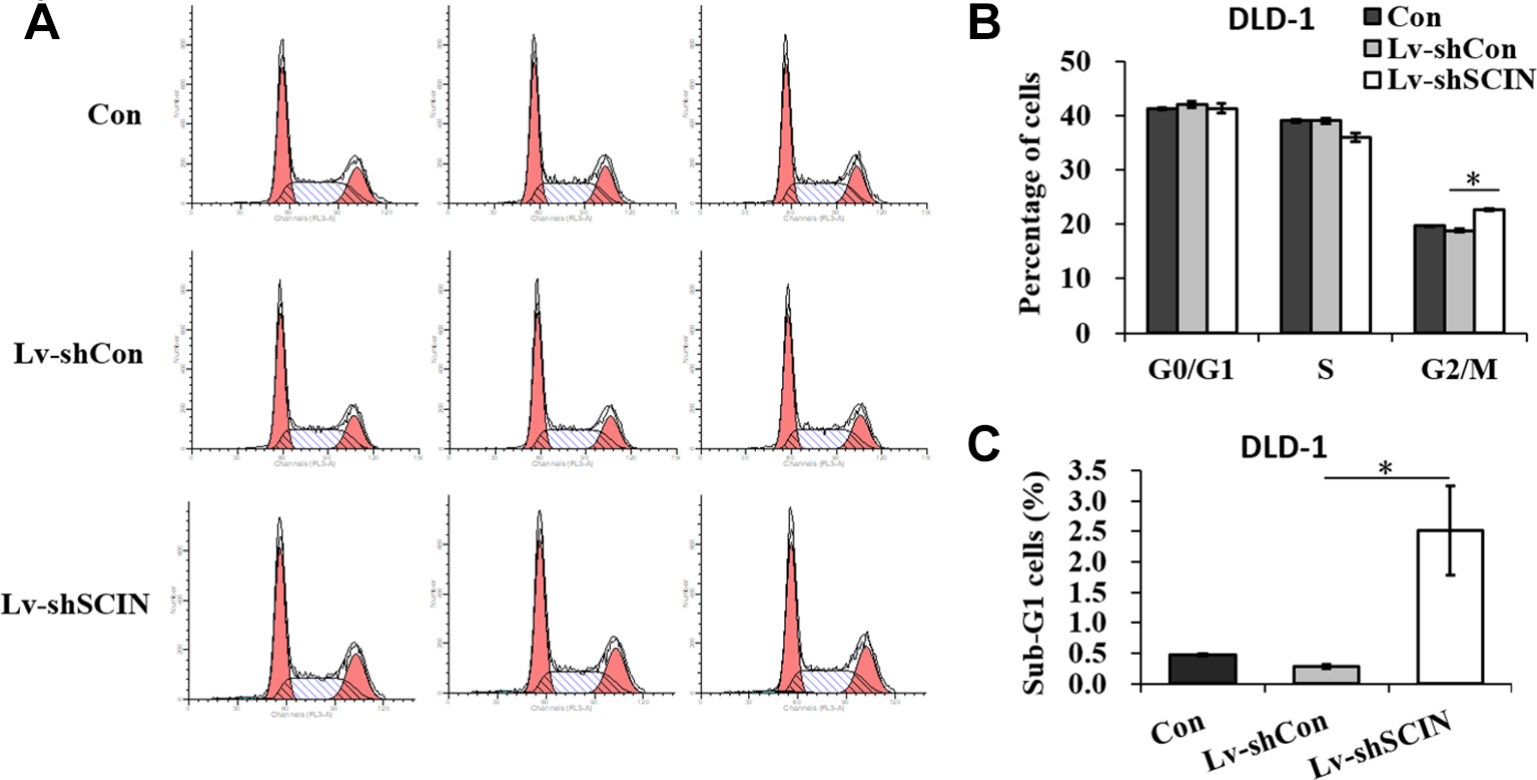
*SCIN* knockdown induces cell cycle arrest. **(A)** Flow cytometry analysis of cell cycle in DLD-1 cells, DLD-1-shSCIN, and DLD-1-shCon cells. **(B)** Statistic results of cell percentages in G0/G1, S, and G2/M phases in DLD-1 cells, DLD-1-shSCIN, and DLD-1-shCon cells. **(C)** Statistic results of cell percentages in the sub-G1 phase in DLD-1 cells, DLD-1-shSCIN, and DLD-1-shCon cells (*P < 0.05).

**Figure 4:**
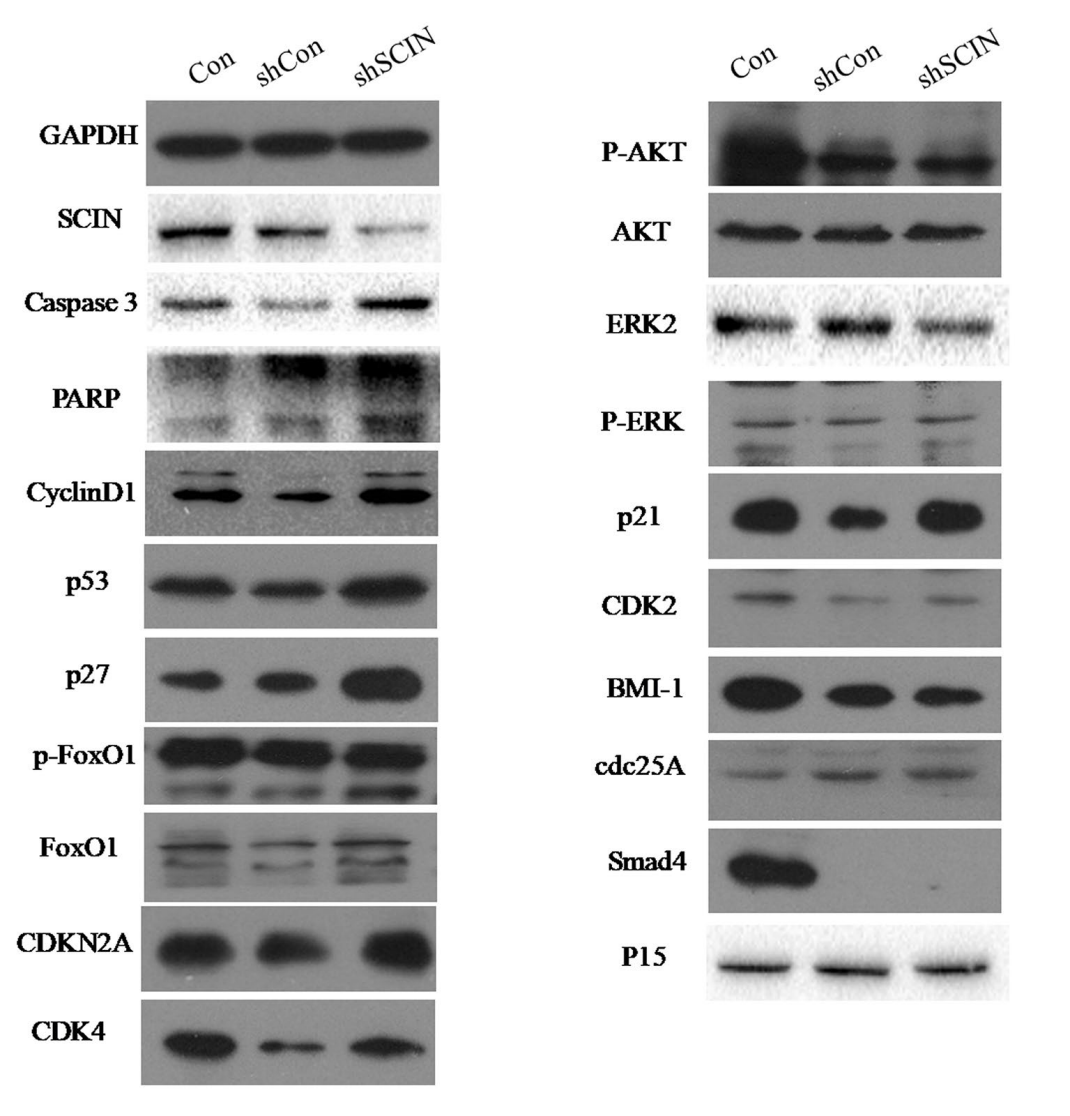
*SCIN* knockdown promoted the expression of some cell cycle apoptosis-related protein. The expression of CyclinD1, P53, P27, p-FoxO1, caspase 3, and PARP in CRC cells was increased by Western blotting analysis. GAPDH was used as a control protein.

Among six CRC cell lines, *SCIN* protein was highly expressed in SW480 and DLD-1 cell lines. So, we used DLD-1 and SW480 cell lines to study the biological function of *SCIN* in cancer cell growth. In order to rule out the potential off-target effect, we also introduced Lv-shSCIN-S1, Lv-shSCIN-S2, and Lv-shSCIN-S3 targeting *SCIN* into DLD-1 cell, and only Lv-shSCIN-S2 inhibited the *SCIN* expression significantly (**Figures 2A–D**). We selected specific *SCIN*-targeting shRNA (S2) for the follow-up experiments, lentivirus mediated RNA interference in DLD-1 and SW480 cell lines were performed. The roles of *SCIN* in cell proliferation was investigated in the DLD-1 and SW480 cell lines using an MTT assay, we found that *SCIN* knockdown significantly reduced cell proliferation (**Figure 2E**). Flow cytometry analysis was used to study the mechanisms by which *SCIN* knockdown induced cell apoptosis. After Lv-shSCIN infection, the DLD-1 cell percentage in the G2/M phase was significantly increased (**Figure 3**). *SCIN* knockdown led to the DLD-1 cell accumulation in the sub-G1 phase, which represents apoptotic cells. The cell cycle markers expression were also increased, including CyclinD1, P53, P27, p-FoxO1, caspase 3, and PARP (**Figure 4**). These results suggested that knockdown of *SCIN* in CRC cells blocked cell cycle progression *via* upregulation of CyclinD1, P53, P27, p-FoxO1, caspase 3, and PARP.

### Other Potential Molecular Mechanisms of *SCIN* Functioning in CRC Cells Analyzed by Microarray Analysis

**FIGURE 5:**
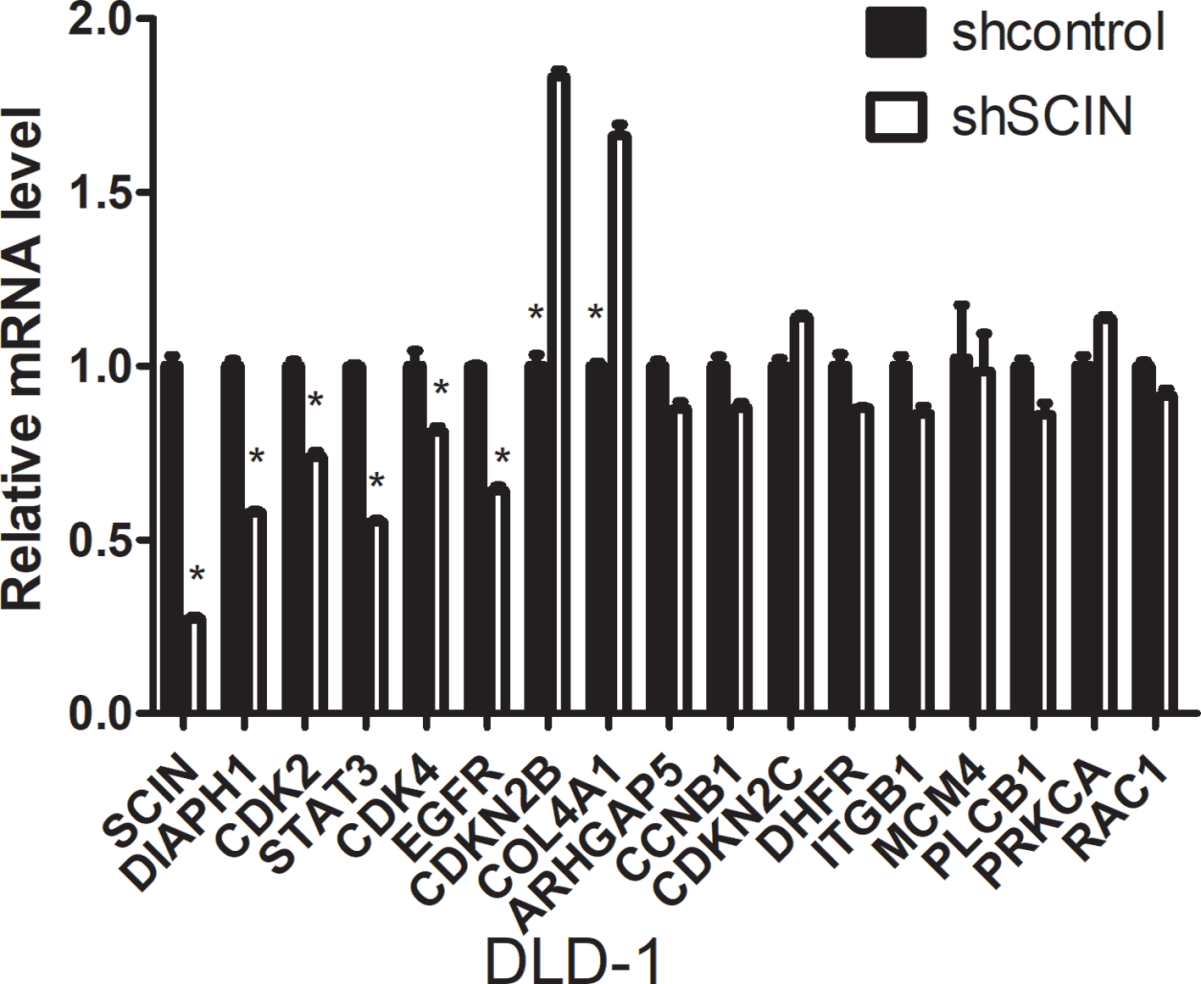
qRT-PCR validation of candidate genes between DLD-1-shSCIN and DLD-1-shCon cells by microarray analysis. Between DLD-1-shSCIN and DLD-1-shCon cells, *DIAPH1*, *CDK2*, *STAT3*, *CDK4*, and *EGFR* RNA were significantly downregulated, whereas *CDKN2B* and *COL4A1* RNA were significantly upregulated. The data shown represent the mean ± s.e.m of the experiment performed in triplicate (*P < 0.05).

Microarray analysis was performed using the Affymetrix GeneChip for DLD-1-shSCIN and DLD-1-shCon cells (the results are listed in **Supplementary Table 4**). Then, we performed gene cluster analysis, GO analysis (molecular function, biological process, and cellular component of MGI GO Slim) and pathway analysis (http://www.broadinstitute.org/gsea/msigdb/annotate.jsp), and the results are listed in **Supplementary Tables 5** and **6**. After comprehensive analysis, we identified 16 genes (*CDKN2C*, *CDKN2B*, *DIAPH1*, *MCM4*, *STAT3*, *EGFR*, *RAC1*, *DHFR*, *CDK2*, *COL4A1*, *PRKCA*, *CDK4*, *CCNB1*, *ARHGAP5*, *ITGB1*, and *PLCB1*) that were significantly correlated with *SCIN* expression. Then, we further examined these differentially expressed genes using qRT-PCR (the primer sequences are shown in **Supplementary Table 7**). We found that between DLD-1-shSCIN and DLD-1-shCon cells, *DIAPH1*, *CDK2*, *STAT3*, *CDK4*, and *EGFR* RNA were significantly downregulated (*P* < 0.05), whereas *CDKN2B* and *COL4A1* RNA were significantly upregulated (*P* < 0.05; **Figure 5**).

## Discussion

CRC is one of the most common malignancies, with an increasing global incidence. Whereas approximately one-half of patients can be cured through surgical resection and adjuvant therapy for well-confined primary tumors, metastatic disease (with the liver as the most common site of CRC metastasis) is largely incurable and is the most frequent cause of mortality due to its systemic nature and the resistance of disseminated tumor cells to existing therapeutic agents (Stein et al., 2009; Valastyan and Weinberg, 2011). The early molecular mechanisms underlying the dynamic and intricate process of metastasis, which are crucial for diagnosis, remain poorly understood (Valastyan and Weinberg, 2011; Budhu and Wang, 2012). Currently, clinical and histopathological findings and tissue-based molecular markers are insufficient for the early identification of individuals at high risk for LM (Duffy et al., 2007).

Very few gene expression profiling studies on CRC have been performed using microarray technology, and these studies have mainly focused on the carcinogenesis process, prognosis prediction, and treatment response prediction (Wang et al., 2004; Arango et al., 2005; Stein et al., 2009), rather than LM in CRC. Gene expression profiling is an innovative and promising approach to investigate underlying molecular mechanisms. Stein et al. (2009) identified a novel gene termed metastasis-associated in colon cancer-1 (*MACC1*), which was found to be associated with colon cancer through genome-wide expression analysis in primary and metastatic carcinomas. The authors demonstrated that *MACC1* expression in tumor specimens served as an independent prognostic indicator of metastasis formation and metastasis-free survival. Wang et al. (2004) used the Affymetrix U133a GeneChip to evaluate RNA samples from 74 patients (31 patients developed tumor relapse in less than 3 years, whereas 43 patients remained disease-free for more than 3 years after surgery). These authors identified a 23-gene signature that predicted recurrence in Dukes’ B patients, which indicated that patients with this 23-gene expression pattern should be upstaged to receive adjuvant therapy, similar to Dukes’ C patients. Arango et al. (2005) performed microarray analysis in a unique set of fresh-frozen tumor samples from Dukes’ C patients who had received surgery as the only form of treatment and found that a high-density oligonucleotide microarray analysis accurately distinguished patients with good and poor prognosis after surgery; in particular, the Ras homologue family member A (*RHOA*) was shown to identify a subset of patients with poor prognosis who could benefit from more aggressive treatment. In the current study, our goal was to identify genes that could predict LM. We applied genome-wide expression analyses in primary tumors with or without LM and identified *SCIN* for the first time. Our study results further suggested that *SCIN* may serve as an important predictor of both LM and poor outcome in CRC.

Although the correlation between *SCIN* and cancer has been revealed, gradually, the role of *SCIN* in tumor development and progression was controversial among different researches. *SCIN* expression in megakaryoblastic leukemia cells promoted cell apoptosis and impaired cell proliferation and tumor formation (Zunino et al., 2001), which is contrary to our study and others (Lai et al., 2018; Qiao et al., 2018). Another study found high levels of *SCIN* expression in gastric cancer tissue correlated with poor prognosis. Furthermore, *SCIN* promoted the invasion and metastasis of gastric cancer cells through activating the Cdc42 pathway to increase the formation of filopodia (Liu et al., 2016). We and other studies found changes of cell cycle distribution affected by *SCIN* (Wang et al., 2014; Liu et al., 2015), but Qiao et al. did not (Qiao et al., 2018). Qiao et al. (2018) and Miura et al. (2012) found that *SCIN* was identified as the cisplatin-resistant marker *via* interacting with VDAC and further inhibiting cell apoptosis in hepatocellular cancer cell and bladder cancer cell, respectively. *SCIN* comprises six homologous domains (A1–A6) that share 60% identity to the six domains from gelsolin (G1–G6), and gelsolin superfamily has been certified to take great role in cell apoptosis by modulating dynamic actin assembly (Kusano et al., 2000). Taken together, the results presented here demonstrated that *SCIN* plays an import role in mediating cytoskeleton reorganization and cell apoptosis.

Based on our preliminary CRC microarray analysis, *SCIN* may affect the diaphanous homolog (*DIAPH1*), cyclin-dependent kinases (*CDK2*), signal transducer and activator of transcription 3 (*STAT3*), cyclin-dependent kinases (*CDK4*), *EGFR*, cycline dependent kinase inhibitor 2B (*CDKN2B*), and collagen type IV alpha 1 (*COL4A1*) signaling pathways. *DIAPH1* is a downstream effector of RhoA (Rho family of GTPases) and controls actin-dependent processes such as cytokinesis, serum response factor transcriptional activity, and cell motility (Narumiya et al., 2009). *DIAPH1* dysregulation within the actin-cytoskeleton pathway may specifically promotes malignant transformation in inflammatory bowel disease-associated CRC (Kanaan et al., 2010). *STAT3* is a transcriptional factor that is constitutively activated in many cancer types. *STAT3* appears to play crucial roles in cell proliferation and survival, angiogenesis, tumor-promoting inflammation, and suppression of antitumor host immune responses in the tumor microenvironment (Yu et al., 2009). *STAT3* activation in CRC is also associated with adverse clinical outcomes, supporting its potential role as a prognostic biomarker and a chemopreventive and/or therapeutic target (Morikawa et al., 2011); although, effectively inhibiting the activity of a transcriptional factor remains challenging due to their intracellular localization and lack of enzymatic activity. However, we found that inhibiting *SCIN* expression could downregulate *STAT3* activity, which implies that anti-*SCIN* therapies may be an effective means to block *STAT3* signaling in CRC. *CDK2* and *CDK4* are extremely important checkpoints of the cell cycle at the G1/S transition. *CDK2* is differentially expressed during colorectal oncogenesis and cancer progression. *CDK2* overexpression may facilitate lymph node metastasis of early cancer, and decreased *CDK2* correlates with large tumor size, venous invasion, deep infiltration, hepatic metastasis, advanced stage, and poor prognosis (Li et al., 2001). *CDK4* overexpression has been detected in intestinal adenomas and is associated with increased cell proliferative activity in premalignant neoplastic cells, which indicates that *CDK4* may contribute to the malignant progression of adenomas (Zhang et al., 1997). *EGFR* belongs to the ERBB receptor tyrosine kinase network, and it plays an important role in CRC pathogenesis. *EGFR* is widely expressed in advanced CRC, with its expression ranging from 72% to 82% (Italiano et al., 2005). Many carcinoma types, including colon, breast, and lung, display heightened EGFR activity, which correlates with tumor recurrence and shorter patient survival (Yarden and Pines, 2012). *CDKN2B* is a cell-cycle inhibitor of INK4/ARF that binds *CDK4* and *CDK6* and induces an allosteric change to abrogate the binding of these kinases to D-type cyclins, thus inhibiting *CDK4/6*-mediated phosphorylation of retinoblastoma (Rb) family members. Studies have demonstrated that *CDKN2B* is deleted in a wide spectrum of tumors including melanoma, pancreatic adenocarcinoma, glioblastoma, certain leukemias, non-small cell lung cancer, and bladder carcinoma (Kim and Sharpless, 2006). In CRC, previous reports have suggested that *CDKN2B* methylation contribute to carcinogenesis along with p16 (INK4a) and that *CDKN2B* may serve as a prognostic factor in the early stages of disease (Ishiguro et al., 2006). Heterotrimers composed of *COL4A1* and *COL4A2* constituted the most abundant components of almost all basement membranes. Mutations in *COL4A1* or *COL4A2* were contribute to a broad spectrum of disorders, including glaucoma, myopathy, and hemorrhagic stroke (Kuo et al., 2012). *COL4A1* was also identified as the potential therapeutic target genes in several human cancers including CRC (Jin et al., 2017).

## Conclusion

Our studies indicate that *SCIN* may serve as an important predictor of CRLM and poor outcome for CRC patients. However, this is a preliminary study, to clarify the molecular mechanism of *SCIN* affection in cell growth and death, and further investigation and more experimental validation was required.

## Ethics Statement

Clinical samples were collected from patients after obtaining written informed consent in accordance with a protocol approved by the Ethics Committee of Zhongshan Hospital of Fudan University (Shanghai, China).

## Author Contributions

QL, JL, YW, and JX conceived the idea and designed the experiments. QL, JL, DZ, and ZN carried out all the experiments and co-wrote the manuscript. XP, PX, and MJ helped with cell culture, PCR, WB, IHC, and FCM. YW and JX supervised the project. All authors read and approved the final manuscript.

## Funding

This study was supported by the National Natural Science Foundation of China (81272390, 81372315, and 81472228), the Shanghai Science and Technology Committee Project (13JC1401601 and 134119a4800), the Shanghai Science and Technology Committee Talent Program (12XD1401900), and the Outstanding Academic Leaders Project of the Health System in Shanghai (XBR2011031).

## Conflict of Interest

The authors declare that the research was conducted in the absence of any commercial or financial relationships that could be construed as a potential conflict of interest.
