# Supplementary material for: Aberrant Scinderin Expression Correlates With Liver Metastasis and Poor Prognosis in Colorectal Cancer
Source: Front Pharmacol. 2019 Oct 31;10:1183. doi: 10.3389/fphar.2019.01183 (PMC6836707; doi:10.3389/fphar.2019.01183)
Supplement: Supplementary file 1 [file DataSheet_1.pdf]

Supplementary Material

Supplemental Tables

Supplementary Table 1: the baseline data of the gene chip patients

number	gender	age	primary tumor site	tumor size (cm)	Pathological type	differentiation	TNM stage
A	male	63	rectum	8	Ulcerative type	moderate	T3N1M1
C	male	52	descending colon	4	Ulcerative type	moderate	T3N1M1
D	male	67	rectum	3	Ulcerative type	moderate	T3N2M1
F	female	42	rectum	5	Ulcerative type	moderate	T4N1M1
E	male	40	Sigmoid colon	4	Ulcerative type	poorly	T4N2M1
B	male	54	Sigmoid colon	4	Ulcerative type	moderate	T4N0M0
H	male	37	ascending colon	5	Ulcerative type	moderate	T3N0M0
I	male	55	rectum	3	Ulcerative type	moderate	T3N0M0
L	male	64	rectum	5	Ulcerative type	moderate	T4N0M0
K	female	68	ascending colon	6	Ulcerative type	moderate	T3N0M0
J	male	29	ascending colon	12	Ulcerative type	poorly	T4N0M0

Supplementary Table 2: the differentially 110 genes by SAM method

ProbeSet	Score	FC	Entrez	Symbol	Gene Title
219312_s_at	4.72	2.32	65986	ZBTB10	zinc finger and BTB domain containing 10
228562_at	4.61	2.27	65986	ZBTB10	Zinc finger and BTB domain containing 10
230573_at	4.54	2.42	10110	SGK2	serum/glucocorticoid regulated kinase 2
208886_at	4.49	2.45	3005	H1F0	H1 histone family, member 0
236557_at	4.33	3.19	253461	ZBTB38	zinc finger and BTB domain containing 38
226064_s_at	4.25	3.15	84649	DGAT2	diacylglycerol O-acyltransferase homolog 2 (mouse)
209691_s_at	4.24	2.76	55715	DOK4	docking protein 4
200632_s_at	4.20	2.0	10397	NDRG1	N-myc downstream regulated gene 1

ProbeSet	Score	FC	Entrez	Symbol	Gene Title
		8			
220349_s_at	4.14	2.07	64772	FLJ21865	endo-beta-N-acetylglucosaminidase
211354_s_at	4.13	3.07	3953	LEPR	leptin receptor
226961_at	4.10	2.80	222171	LOC222171	hypothetical protein LOC222171
1555467_a_at	4.00	2.72	10658	CUGBP1	CUG triplet repeat, RNA binding protein 1
244261_at	4.00	2.20	163702	IL28RA	interleukin 28 receptor, alpha (interferon, lambda receptor)
233899_x_at	3.97	2.45	65986	ZBTB10	Zinc finger and BTB domain containing 10
230784_at	3.88	5.39	84366	PRAC	small nuclear protein PRAC
220161_s_at	3.83	2.21	54566	EPB41L4B	erythrocyte membrane protein band 4.1 like 4B
206286_s_at	3.80	2.87	6997	TDGF1	teratocarcinoma-derived growth factor 1
236518_at	3.78	2.69	84960	KIAA1984	KIAA1984
219215_s_at	3.70	2.97	55630	SLC39A4	solute carrier family 39 (zinc transporter), member 4
229986_at	3.68	2.17	377064	LOC377064	kruppel-like zinc finger factor X17
225177_at	3.56	2.02	80223	RAB11FIP1	RAB11 family interacting protein 1 (class I)
218345_at	3.48	2.29	55365	HCA112	hepatocellular carcinoma-associated antigen 112
208510_s_at	3.48	2.54	5468	PPARG	peroxisome proliferative activated receptor, gamma
1552334_at	3.38	2.63	11078	TRIOBP	TRIO and F-actin binding protein
228876_at	3.38	2.13	80115	BAIAP2L2	BAI1-associated protein 2-like 2
1555829_at	3.33	2.48	57488	FAM62B	family with sequence similarity 62 (C2 domain containing) member B
214848_at	3.28	2.02	7534	YWHAZ	Tyrosine 3-monooxygenase/tryptophan 5-monooxygenase activation protein, zeta polypeptide
229377_at	3.18	2.00	79774	GRTP1	Growth hormone regulated TBC protein 1
202314_at	3.16	2.18	1595	CYP51A1	cytochrome P450, family 51, subfamily A, polypeptide 1
1552365_at	3.15	2.55	85477	SCIN	scinderin

ProbeSet	Score	FC	Entrez	Symbol	Gene Title
205593_s_at	3.15	6.67	5152	PDE9A	phosphodiesterase 9A
203953_s_at	3.14	3.25	1365	CLDN3	claudin 3
241547_at	3.13	2.27	---	---	MRNA; cDNA DKFZp779G2222 (from clone DKFZp779G2222)
220951_s_at	3.13	2.25	29974	ACF	apobec-1 complementation factor
203954_x_at	3.10	2.59	1365	CLDN3	claudin 3
44790_s_at	3.07	2.53	80183	C13orf18	chromosome 13 open reading frame 18
213435_at	3.06	2.29	23314	SATB2	SATB family member 2
213324_at	3.01	2.35	6714	SRC	v-src sarcoma (Schmidt-Ruppin A-2) viral oncogene homolog (avian)
239859_x_at	2.97	2.90	8814	CDKL1	Cyclin-dependent kinase-like 1 (CDC2-related kinase)
1555788_a_at	2.97	2.41	57761	TRIB3	tribbles homolog 3 (Drosophila)
218510_x_at	2.96	2.00	54463	FLJ20152	hypothetical protein FLJ20152
229358_at	2.96	2.18	3549	IHH	Indian hedgehog homolog (Drosophila)
219471_at	2.94	2.19	80183	C13orf18	chromosome 13 open reading frame 18
227371_at	2.93	2.05	55971	BAIAP2L1	BAI1-associated protein 2-like 1
242604_at	2.85	2.48	282679	AQP11	Aquaporin 11
223423_at	2.82	2.52	26996	GPR160	G protein-coupled receptor 160
221020_s_at	2.79	2.02	81034	SLC25A32	solute carrier family 25, member 32 /// solute carrier family 25, member 32
228302_x_at	2.76	2.32	55450	CAMK2N1	calcium/calmodulin-dependent protein kinase II inhibitor 1
203628_at	2.75	2.14	3480	IGF1R	insulin-like growth factor 1 receptor
213308_at	2.75	2.30	22941	SHANK2	SH3 and multiple ankyrin repeat domains 2
227845_s_at	2.74	2.42	56961	SHD	Src homology 2 domain containing transforming protein D
209051_s_at	2.74	2.31	5900	RALGDS	ral guanine nucleotide dissociation stimulator
225727_at	2.73	2.63	57475	PLEKHH1	pleckstrin homology domain containing, family H (with MyTH4 domain) member 1

ProbeSet	Score	FC	Entrez	Symbol	Gene Title
209309_at	2.71	2.68	563	AZGP1	alpha-2-glycoprotein 1, zinc
205081_at	2.71	2.44	1396	CRIP1	cysteine-rich protein 1 (intestinal)
242871_at	2.70	2.65	54852	PAQR5	progestin and adipoQ receptor family member V
206268_at	2.70	2.90	10637	LEFTY1	left-right determination factor 1
218814_s_at	2.70	2.05	55248	C1orf75	chromosome 1 open reading frame 75
210808_s_at	2.70	2.92	27035	NOX1	NADPH oxidase 1
206992_s_at	2.70	2.03	27109	ATP5S	ATP synthase, H+ transporting, mitochondrial F0 complex, subunit s (factor B)
215986_at	-4.34	0.40	---	---	CDNA FLJ12058 fis, clone HEMBB1002092
1559618_at	-4.33	0.43	1840	DTX1	Deltex homolog 1 (Drosophila)
1555153_s_at	-4.03	0.49	115548	FCHO2	FCH domain only 2
218935_at	-3.94	0.43	30845	EHD3	EH-domain containing 3
227474_at	-3.77	0.42	7849	PAX8	Paired box gene 8
239596_at	-3.74	0.49	148867	SLC30A7	solute carrier family 30 (zinc transporter), member 7
207375_s_at	-3.72	0.42	3601	IL15RA	interleukin 15 receptor, alpha
1555421_at	-3.66	0.30	130340	AP1S3	adaptor-related protein complex 1, sigma 3 subunit
206340_at	-3.59	0.47	9971	NR1H4	nuclear receptor subfamily 1, group H, member 4
212624_s_at	-3.57	0.40	1123	CHN1	chimerin (chimaerin) 1
238050_at	-3.55	0.28	---	---	Transcribed locus
239418_x_at	-3.50	0.49	404033	FLJ34077	Weakly similar to zinc finger protein 195
221261_x_at	-3.46	0.46	81557	MAGED4	melanoma antigen family D, 4 /// melanoma antigen family D, 4
210115_at	-3.46	0.50	116832	RPL39L	ribosomal protein L39-like
238790_at	-3.43	0.25	374443	LOC374443	CLR pseudogene
1564670_at	-3.41	0.45	10266	RAMP2	Receptor (calcitonin) activity modifying protein 2

ProbeSet	Score	FC	Entrez	Symbol	Gene Title
205667_at	-3.31	0.49	7486	WRN	Werner syndrome
229327_s_at	-3.30	0.41	4094	MAF	V-maf musculoaponeurotic fibrosarcoma oncogene homolog (avian)
207709_at	-3.24	0.41	5563	PRKAA2	protein kinase, AMP-activated, alpha 2 catalytic subunit
206455_s_at	-3.16	0.27	6010	RHO	rhodopsin (opsin 2, rod pigment) (retinitis pigmentosa 4, autosomal dominant)
238617_at	-3.16	0.44	---	---	CDNA FLJ38181 fis, clone FCBBF1000125
241866_at	-3.15	0.42	9194	SLC16A7	solute carrier family 16 (monocarboxylic acid transporters), member 7
238584_at	-3.12	0.26	79781	IQCA	IQ motif containing with AAA domain
219983_at	-3.11	0.37	57110	HRASLS	HRAS-like suppressor
211863_x_at	-3.07	0.42	3077	HFE	hemochromatosis
212921_at	-3.07	0.48	56950	SMYD2	SET and MYND domain containing 2
237602_at	-3.06	0.44	57554	LRRC7	Leucine rich repeat containing 7
244427_at	-3.04	0.49	9493	KIF23	Kinesin family member 23
1560526_at	-3.01	0.39	266671	PR47	platelet receptor for type III collagen, 47 kDa
1555912_at	-3.00	0.40	93653	ST7OT1	ST7 overlapping transcript 1 (antisense non-coding RNA)
232453_at	-2.99	0.14	10082	GPC6	Glypican 6
234472_at	-2.92	0.46	114805	GALNT13	UDP-N-acetyl-alpha-D-galactosamine:polypeptide N-acetylgalactosaminyltransferase 13 (GalNAc-T13)
206618_at	-2.91	0.24	8809	IL18R1	interleukin 18 receptor 1
219454_at	-2.89	0.39	25975	EGFL6	EGF-like-domain, multiple 6
237376_at	-2.88	0.46	8452	CUL3	Cullin 3
1554157_a_at	-2.86	0.46	90199	WFDC8	WAP four-disulfide core domain 8
225316_at	-2.84	0.43	84879	MFSD2	major facilitator superfamily domain containing 2
205715_at	-2.82	0.30	683	BST1	bone marrow stromal cell antigen 1
243506_at	-2.81	0.3	---	---	Transcribed locus

ProbeSet	Score	FC	Entrez	Symbol	Gene Title
		4			
211866_x_at	-2.79	0.48	3077	HFE	hemochromatosis
204055_s_at	-2.78	0.35	4253	CTAGE5	CTAGE family, member 5
206104_at	-2.76	0.21	3670	ISL1	ISL1 transcription factor, LIM/homeodomain, (islet-1)
202952_s_at	-2.75	0.35	8038	ADAM12	ADAM metallopeptidase domain 12 (meltrin alpha)
214341_at	-2.74	0.49	8906	AP1G2	adaptor-related protein complex 1, gamma 2 subunit
218484_at	-2.74	0.46	56901	LOC56901	NADH:ubiquinone oxidoreductase MLRQ subunit homolog
1559631_at	-2.73	0.44	---	---	Homo sapiens, clone IMAGE:4045663, mRNA
1570021_at	-2.73	0.41	360030	LOC360030	homeobox C14
230715_at	-2.72	0.37	85460	KIAA1729	KIAA1729 protein
242171_at	-2.72	0.42	---	---	---
213309_at	-2.70	0.50	23228	PLCL2	phospholipase C-like 2

Supplementary Table 3: the baseline data of the 60 patients examined by qRT-PCR

	CRC without LM	CRC with SLM	CRC with MLM
Age	59.6±12.3	56.5±10.7	60.8±12.9
Gender			
male	12	13	9
female	8	7	11
Primary tumor site			
colon	10	13	13
rectum	10	7	7
Tumor diameter	5.05 ± 1.31	5.95 ± 1.50	6.10 ± 2.81
T stage			
T 0-2	1	0	0
T 3	3	0	5
T4	16	20	15
N stage			
N0	16	4	6
N1	3	9	6
N2	1	7	8

Supplementary Table 4: The results of the Microarray analysis of DLD-1-shSCIN and shCon cells (257 genes upregulated, 695 genes downregulated)

Probe Set ID	Gene Symbol	Entrez Gene	Alignments	Regulation([KD] vs [NC])
11732684_a_at	ABCA12	26154	chr2:215796266-216003151 (-) // 99.41 // q35	up
11730907_a_at	ABCB1	5243	chr7:87132339-87342727 (-) // 96.55 // q21.12	up
11724538_a_at	ABCG2	9429	chr4:89011416-89080214 (-) // 86.41 // q22.1	up
11746956_a_at	ABHD2	11057	chr15:89631710-89745591 (+) // 91.33 // q26.1	up
11742135_at	ACBD4	79777	chr17:43213013-43216527 (+) // 74.87 // q21.31	up
11721065_at	ADCY9	115	chr16:4012649-4166186 (-) // 96.37 // p13.3	up
11729003_at	ADRB1	153	chr10:115803811-115806955 (+) // 92.4 // q25.3	up
11754888_a_at	ADSSL1	122622	chr14:105210817-105213645 (+) // 84.06 // q32.33	up
11720421_at	AFF4	27125	chr5:132211075-132299354 (-) // 98.62 // q31.1	up
11717131_a_at	AHNAK2	113146	chr14:105403589-105444694 (-) // 99.47 // q32.33	up
11715711_a_at	AKR1C3	8644	chr10:5135984-5149878 (+) // 93.02 // p15.1	up
11757588_a_at	ALG5	29880	chr13:37523980-37527163 (-) // 95.54 // q13.3	up
11744583_s_at	AMDHD2	51005	chr16:2570365-2579732 (+) // 99.19 // p13.3	up
11757989_s_at	ANKRD46	157567	chr8:101533001-101533436 (-) // 96.67 // q22.2	up
11740603_a_at	APH1B	83464	chr15:63569748-63601324 (+) // 92.43 // q22.2	up
11715428_x_at	ARF4	378	chr3:57557089-57583589 (-) // 80.35 // p14.3	up
11750222_a_at	ARID5B	84159	chr10:63661446-63811107 (+) // 99.92 // q21.2	up
11716707_a_at	ARL2BP	23568	chr16:57279009-57287547 (+) // 95.14 // q13	up
11746863_x_at	ARSJ	79642	chr4:114821399-114900857 (-) // 93.05 // q26	up
11746932_a_at	ASPHD1	253982	chr16:29912178-29931183 (+) // 78.21 // p11.2	up
11729305_a_at	ATAD1	84896	chr10:89512874-89577950 (-) // 93.12 // q23.31	up
11719344_a_at	ATF3	467	chr1:212781993-212794117 (+) // 99.08 // q32.3	up
11717801_a_at	ATF5	22809	chr19:50431958-50437191 (+) // 88.96 // q13.33	up
11722183_s_at	ATP6V1C1	528	chr8:104033244-104085912 (+) // 82.41 // q22.3	up

11754004_a_at	ATP6V1G1	9550	chr9:117349842-117360140 (+) // 90.78 // q32	up
11748950_a_at	BAZ2B	29994	chr2:160255351-160472028 (-) // 94.96 // q24.2	up
11732337_x_at	BCAS1	8537	chr20:52560082-52687374 (-) // 90.81 // q13.2	up
11724673_a_at	BCKDHB	594	chr6:80816343-81055987 (+) // 98.54 // q14.1	up
11740374_a_at	BCL2L14	79370	chr12:12223872-12252625 (+) // 97.83 // p13.2	up
11731176_at	BCL2L15	440603	chr1:114419611-114430203 (-) // 63.29 // p13.2	up
11716823_s_at	BDH2	56898	chr6:99622547-99625462 (+) // 66.58 // q16.2 /// chr4:103998764-104021064 (-) // 75.83 // q24	up
11716822_a_at	BDH2 /// NHEDC2	133308 /// 56898	chr6:99622547-99625462 (+) // 66.58 // q16.2 /// chr4:103998764-104021064 (-) // 75.83 // q24	up
11763406_s_at	BICC1	80114	chr10:60588982-60591183 (+) // 98.52 // q21.1	up
11722953_a_at	BIK	638	chr22:43506753-43525718 (+) // 90.79 // q13.2	up
11717008_a_at	BNIP3L	665	chr8:26240413-26270641 (+) // 97.27 // p21.2	up
11723422_s_at	BTF3L4	91408	chr1:52521801-52554585 (+) // 87.15 // p32.3 /// chr2:159860161-159861230 (+) // 33.65 // q24.2 /// chr21:18889276-18891379 (-) // 69.07 // q21.1	up
11723420_s_at	BTF3L4 /// LOC100505945	100505945 /// 91408	chr1:52521801-52554585 (+) // 87.15 // p32.3 /// chr2:159860161-159861230 (+) // 33.65 // q24.2 /// chr21:18889276-18891379 (-) // 69.07 // q21.1	up
11724786_s_at	C14orf129	51527	chr14:96830716-96853623 (+) // 83.97 // q32.2	up
11736067_a_at	C15orf63 /// SERF2	10169 /// 25764	chr15:44092348-44094767 (+) // 80.41 // q15.3	up
11736916_a_at	C17orf58	284018	chr17:65987217-65989765 (-) // 85.22 // q24.2	up
11736304_a_at	C17orf91	84981	chr17:1615081-1619504 (-) // 43.15 // p13.3	up
11742699_a_at	C1R	715	chr12:7187512-7245065 (-) // 89.76 // p13.31	up
11741160_a_at	C1orf26	54823	chr1:185126290-185260912 (+) // 99.07 // q25.3	up
11756435_a_at	C1orf85	112770	chr1:156262486-156265468 (-) // 98.24 // q22	up
11754909_a_at	C2orf88	84281	chr2:191045164-191064935 (+) //	up

			95.76 // q32.2	
11728449_at	C3orf57	165679	chr3:161062583-161090668 (-) // 89.23 // q26.1	up
11734497_at	C6orf222	389384	chr6:36283533-36304662 (-) // 83.29 // p21.31	up
11723151_s_at	C7orf10	79783	chr7:40174480-40900357 (+) // 87.42 // p14.1	up
11721984_at	C7orf29	113763	chr7:150026875-150029810 (+) // 95.13 // q36.1	up
11751867_a_at	C8orf59	401466	chr8:86129391-86132569 (-) // 77.09 // q21.2	up
11721568_a_at	C9orf3	84909	chr9:97488714-97849500 (+) // 80.89 // q22.32	up
11754856_a_at	CA12	771	chr15:63616905-63674075 (-) // 96.41 // q22.2	up
11723517_a_at	CAB39L	81617	chr13:49882785-49975735 (-) // 99.35 // q14.2	up
11758576_s_at	CCDC59	29080	chr12:82746089-82746492 (-) // 38.73 // q21.31	up
11724828_at	CCL20	6364	chr2:228678557-228682272 (+) // 95.97 // q36.3	up
11755103_a_at	CDA	978	chr1:20915440-20945385 (+) // 84.46 // p36.12	up
11729118_a_at	CDKN1C	1028	chr11:2904447-2907111 (-) // 79.41 // p15.4	up
11736163_a_at	CDKN2B	1030	chr9:22002902-22009385 (-) // 92.07 // p21.3	up
11739039_x_at	CEACAM5	1048	chr19:42212503-42628368 (+) // 63.96 // q13.2	up
11725980_at	CFD	1675	chr19:859452-863750 (+) // 72.29 // p13.3	up
11718118_s_at	CFL2	1073	chr14:35179587-35183906 (-) // 96.82 // q13.1	up
11724885_at	CLIC3	9022	chr9:139889082-139891017 (-) // 91.69 // q34.3	up
11754675_a_at	CLIP4	79745	chr2:29337743-29397580 (+) // 95.92 // p23.2	up
11716638_s_at	COL4A1	1282	chr13:110801310-110959496 (-) // 99.04 // q34	up
11729128_at	CPA4	51200	chr7:129932973-129964014 (+) // 85.97 // q32.2	up
11730761_s_at	CRIPT	9419	chr2:46844289-46852883 (+) // 77.97 // p21	up
11722981_a_at	CSPG5	10675	chr3:47603728-47622282 (-) // 91.86 // p21.31	up
11734776_a_at	CTTN	2017	chr11:70244635-70282681 (+) // 97.33 // q13.3	up
11758000_s_at	CXADR	1525	chr21:18938820-18939264 (+) // 97.16 // q21.1 /// chr21:15050246-15050713 (+) // 92.12 // q11.2 /// chr15:22014422-22014890 (-) // 91.68 // q11.2 ///	up

11753586_s_at	CXADR /// CXADRP2 /// CXADRP3	1525 /// 440224 /// 646243	chr15:21007759-21008227 (-) // 91.03 // q11.2 /// chr18:14476890-14477357 (-) // 91.25 // p11.21 chr21:18885447-18937933 (+) // 99.83 // q21.1 /// chr21:15048342-15049360 (+) // 93.86 // q11.2 /// chr15:21009115-21010134 (-) // 95.69 // q11.2 /// chr15:22015778-22016796 (-) // 95.19 // q11.2 /// chr18:14478243-14479266 (-) // 95.36 // p11.21	up
11719366_s_at	CXCL1	2919	chr4:74735118-74737019 (+) // 97.29 // q13.3	up
11744128_x_at	CXCL2	2920	chr4:74962676-74965002 (-) // 94.46 // q13.3	up
11728477_at	CXCL3	2921	chr4:74902305-74904523 (-) // 91.73 // q13.3	up
11750061_x_at	DAZAP2	9802	chr12:51632607-51636608 (+) // 99.32 // q13.13 /// chr2:203065740-203066692 (-) // 93.78 // q33.1	up
11719738_at	DCP2	167227	chr5:112312398-112356761 (+) // 96.56 // q22.2	up
11723899_a_at	DHRS9	10170	chr2:169921298-169952677 (+) // 85.44 // q31.1	up
11724848_a_at	DIXDC1	85458	chr11:111807926-111893374 (+) // 93.97 // q23.1	up
11727900_at	DMRT1	1761	chr9:841644-969090 (+) // 96.33 // p24.3	up
11737097_a_at	DSC2	1824	chr18:28645943-28682388 (-) // 96.68 // q12.1	up
11753617_a_at	DUSP3	1845	chr17:41846976-41856292 (-) // 100.0 // q21.31	up
11724575_at	ECE2	9718	chr3:183967385-183977406 (+) // 93.77 // q27.1	up
11726023_a_at	EDN1	1906	chr6:12290311-12297426 (+) // 98.8 // p24.1	up
11718830_s_at	EHD1	10938	chr11:64620202-64646230 (-) // 96.8 // q13.1	up
11717594_s_at	ELF3	1999	chr1:201979689-201986311 (+) // 91.6 // q32.1	up
11759423_at	EPPK1	83481	chr8:144939496-144947470 (-) // 49.29 // q24.3	up
11758563_at	FAM119A	151194	chr2:208477464-208477880 (-) // 98.81 // q33.3	up
11739380_a_at	FAM198B	51313	chr4:159045625-159093888 (-) // 98.11 // q32.1	up
11737653_a_at	FAM26D	221301	chr6:116850194-116880031 (+) // 84.83 // q22.1	up
11722236_at	FBXO2	26232	chr1:11708417-11714890 (-) // 83.32	up

			// p36.22	
11739518_a_at	FLRT3	23767	chr20:14303922-14318313 (-) // 83.23 // p12.1	up
11717930_x_at	FOLR1	2348	chr11:71901257-71907345 (+) // 96.59 // q13.4	up
11716131_s_at	FSTL3	10272	chr19:676390-683484 (+) // 96.51 // p13.3	up
11732322_at	FXN	2395	chr9:71650174-71689099 (+) // 73.82 // q21.11	up
11715479_a_at	GABARAP	11337	chr17:7143388-7146437 (-) // 97.64 // p13.1	up
11728763_x_at	GAL3ST4	79690	chr7:99756866-99766373 (-) // 85.82 // q22.1	up
11748926_a_at	GANAB	23193	chr11:62392903-62407205 (-) // 89.75 // q12.3	up
11716814_s_at	GATM	2628	chr15:45653321-45672321 (-) // 80.14 // q21.1	up
11730481_a_at	GJB6	10804	chr13:20796101-20806732 (-) // 96.35 // q12.11	up
11715721_a_at	GOLM1	51280	chr9:88640937-88714502 (-) // 94.82 // q21.33	up
11731538_a_at	GPR110	266977	chr6:46967812-47010099 (-) // 97.04 // p12.3	up
11742775_a_at	GPR161	23432	chr1:168053321-168105624 (-) // 91.29 // q24.2	up
11737992_s_at	GPR37	2861	chr7:124386115-124405681 (-) // 96.63 // q31.33	up
11719762_a_at	GPSM1	26086	chr9:139221931-139254052 (+) // 93.15 // q34.3	up
11754760_a_at	GRAMD1A	57655	chr19:35485687-35517375 (+) // 97.25 // q13.11	up
11745074_a_at	GSTA4	2941	chr6:52842753-52850383 (-) // 75.3 // p12.2	up
11722903_s_at	GSTT2 /// GSTT2B	2953 /// 653689	chr22:24322338-24326106 (+) // 68.89 // q11.23 /// chr22:24299600-24303368 (-) // 68.82 // q11.23	up
11752897_a_at	HBP1	26959	chr7:106809447-106842011 (+) // 100.0 // q22.3	up
11721473_a_at	HCCS	3052	chrX:11129417-11141195 (+) // 91.07 // p22.2	up
11736244_s_at	HIST1H2AC	8334	chr6:26124396-26138605 (+) // 90.6 // p22.2	up
11738058_s_at	HIST1H2AD	3013	chr6:26198964-26199521 (-) // 100.0 // p22.2	up
11741704_x_at	HIST1H2BC	8347	chr6:26123583-26124154 (-) // 100.0 // p22.2	up
11759760_s_at	HIST1H2BC /// LOC100506979	100506979 /// 8347	chr6:26115113-26124154 (-) // 84.91 // p22.2	up
11724021_at	HIST1H2BD	3017	chr6:26158348-26171577 (+) // 96.22 // p22.2	up
11741186_at	HIST1H3D	8351	chr6:26196982-26199521 (-) // 77.41 // p22.2	up

11742688_s_at	HIST2H2AA3 /// HIST2H2AA4	723790 /// 8337	chr1:149822467-149823195 (+) // 88.54 // q21.2 /// chr1:149813750-149814478 (-) // 88.54 // q21.2	up
11717090_a_at	HIST2H2BE	8349	chr1:149856011-149859515 (-) // 87.27 // q21.2	up
11742669_s_at	HIST2H4A /// HIST2H4B	554313 /// 8370	chr1:149804220-149804616 (+) // 100.0 // q21.2 /// chr1:149832329-149832725 (-) // 100.0 // q21.2	up
11744829_s_at	HLA-E	3133	chr6:30457289-30460789 (+) // 79.68 // p21.33	up
11759794_at	HSD17B12	51144	chr11:43702277-43776952 (+) // 39.15 // p11.2	up
11739252_x_at	HSD3B1	3283	chr1:120049832-120057681 (+) // 97.55 // p12	up
11717903_s_at	IER3IP1	51124	chr18:44681412-44702735 (-) // 93.75 // q21.1	up
11729742_x_at	IFI27L2	83982	chr14:94594132-94595986 (-) // 84.83 // q32.12	up
11755606_x_at	IFI30	10437	chr19:18288661-18288927 (+) // 95.34 // p13.11	up
11721874_at	IFIT2	3433	chr10:91061522-91069032 (+) // 97.51 // q23.31	up
11718025_at	IL1R1	3554	chr2:102721033-102796334 (+) // 93.64 // q12.1	up
11754026_a_at	IL8	3576	chr4:74606280-74608177 (+) // 70.26 // q13.3	up
11719141_a_at	INSIG2	51141	chr2:118845998-118868679 (+) // 81.89 // q14.2	up
11724668_at	INSL4	3641	chr9:5231401-5235528 (+) // 89.08 // p24.1	up
11720209_at	IRF9	10379	chr14:24630261-24635774 (+) // 96.44 // q12	up
11756806_a_at	ISG20	3669	chr15:89182193-89198847 (+) // 44.44 // q26.1	up
11719292_a_at	ITGB1BP1	9270	chr2:9545809-9563695 (-) // 98.95 // p25.1	up
11725806_at	KCNJ8	3764	chr12:21917889-21927754 (-) // 99.13 // p12.1	up
11736458_x_at	KCNK6	9424	chr19:38810468-38819553 (+) // 56.19 // q13.2	up
11731580_x_at	KCTD20	222658	chr6:36410543-36458918 (+) // 85.4 // p21.31	up
11719928_a_at	KDM5B	10765	chr1:202696344-202778598 (-) // 90.96 // q32.1	up
11731363_a_at	KLC3	147700	chr19:45843997-45854778 (+) // 93.68 // q13.32	up
11729691_a_at	KLHL24	54800	chr3:183353355-183402302 (+) // 90.88 // q27.1	up
11732196_x_at	KPNA4	3840	chr3:160217420-160283376 (-) // 97.08 // q25.33	up
11722835_a_at	KPTN	11133	chr19:47978406-47987531 (-) //	up

			89.22 // q13.32	
11728308_at	KRT20	54474	chr17:39032147-39041495 (-) // 97.93 // q21.2	up
11738301_at	KRTAP3-1	83896	chr17:39164775-39165366 (-) // 96.25 // q21.2	up
11738427_at	KRTAP4-1	85285	chr17:39340356-39341600 (-) // 91.76 // q21.2	up
11746635_a_at	LEF1	51176	chr4:108969365-109088923 (-) // 93.75 // q25	up
11721237_a_at	LHFPL2	10184	chr5:77781052-77944648 (-) // 95.8 // q14.1	up
11760084_x_at	LOC254896	254896	chr8:22941867-22961063 (+) // 77.45 // p21.3	up
11746708_at	LOC389834	389834	chr2:95526395-95532937 (+) // 68.15 // q11.1 /// chr21:9916851-9923445 (-) // 72.41 // p11.2 /// chr4_gl000194_random:63252-69842 (-) // 71.99 // /// chrY:13319117-13325698 (-) // 71.04 // q11.1	up
11763340_s_at	LOC642852	642852	chr21:46714863-46716111 (+) // 31.59 // q22.3	up
11746283_s_at	LOC730755	730755	chr17:39215494-39216366 (-) // 94.12 // q21.2	up
11717464_at	LXN	56925	chr3:158383817-158390482 (-) // 96.82 // q25.32	up
11728507_at	LY96	23643	chr8:74903586-74941314 (+) // 77.76 // q21.11	up
11733938_x_at	LYPLA2	11313	chr1:24117624-24122027 (+) // 96.62 // p36.11 /// chr6:33332515-33334135 (-) // 92.53 // p21.32	up
11745723_a_at	MALAT1	378938	chr11:65265232-65273937 (+) // 94.71 // q13.1	up
11739372_a_at	MAPRE2	10982	chr18:32558207-32723430 (+) // 98.8 // q12.1	up
11724471_x_at	MGST1	4257	chr12:16500587-16517400 (+) // 88.42 // p12.3	up
11755719_a_at	MLLT11	10962	chr1:151032880-151040969 (+) // 99.93 // q21.3	up
11743691_a_at	MRPL30	51263	chr2:99797577-99814089 (+) // 99.4 // q11.2	up
11723056_a_at	MRPL33	9553	chr2:27994583-28002601 (+) // 79.51 // p23.2	up
11726608_s_at	MTF2	22823	chr1:93544794-93604631 (+) // 98.38 // p22.1	up
11724833_at	MUC13	56667	chr3:124624288-124646840 (-) // 93.49 // q21.2	up
11754299_a_at	MXD4	10608	chr4:2249158-2258477 (-) // 91.34 // p16.3	up
11738617_s_at	MYO7A	4647	chr11:76839315-76926284 (+) // 99.58 // q13.5	up
11725335_s_at	MZT1	440145	chr13:73282504-73302001 (-) //	up

			96.63 // q21.33	
11745183_a_at	NAA15	80155	chr4:140222654-140310342 (+) // 91.62 // q31.1	up
11743415_s_at	NDRG4	65009	chr16:58497769-58547522 (+) // 93.27 // q21	up
11757513_at	NFKBIZ	64332	chr3:101579157-101580117 (+) // 80.46 // q12.3	up
11735764_a_at	NOSTRIN	115677	chr2:169659127-169721564 (+) // 98.31 // q24.3	up
11718965_s_at	NUFIP2	57532	chr17:27582857-27621166 (-) // 94.03 // q11.2	up
11722015_at	OLR1	4973	chr12:10310899-10324802 (-) // 78.55 // p13.2	up
11728681_s_at	PAIP1	10605	chr17:18553507-18556020 (+) // 84.88 // p11.2 /// chr5:43526368-43557195 (-) // 96.94 // p12	up
11722232_a_at	PCP4	5121	chr21:41239242-41301322 (+) // 95.92 // q22.2	up
11723499_a_at	PCSK9	255738	chr1:55505065-55530525 (+) // 92.78 // p32.3	up
11718305_a_at	PDLIM2	64236	chr8:22436253-22451811 (+) // 96.25 // p21.3	up
11759721_a_at	PDXK	8566	chr21:45148780-45158741 (+) // 58.96 // q22.3	up
11724779_at	PGM2L1	283209	chr11:74041358-74109799 (-) // 85.87 // q13.4	up
11756532_a_at	PIK3CD	5293	chr1:9711788-9788969 (+) // 79.63 // p36.22	up
11715880_a_at	PITPNA	5306	chr17:1421133-1466110 (-) // 94.49 // p13.3	up
11730699_at	PLA2G10	8399	chr16:14766406-14788526 (-) // 88.92 // p13.12 /// chr16:14821137-14827644 (-) // 69.41 // p13.11	up
11728421_a_at	PLA2G7	7941	chr6:46671941-46703153 (-) // 95.83 // p12.3	up
11728184_a_at	PLEKHA7	144100	chr11:16809030-17035963 (-) // 97.85 // p15.1	up
11720452_at	PLEKHH2	130271	chr2:43864437-43995126 (+) // 91.53 // p21	up
11746264_a_at	PPAP2A	8611	chr5:54720998-54830503 (-) // 98.62 // q11.2	up
11763834_a_at	PPP1R1C	151242	chr2:182968327-182982644 (+) // 97.53 // q31.3	up
11752589_a_at	PPYR1	5540	chr10:47083533-47088320 (+) // 99.89 // q11.22	up
11728764_a_at	PVRL4	81607	chr1:161040780-161059385 (-) // 93.16 // q23.3	up
11724800_a_at	QPRT	23475	chr16:29690439-29709610 (+) // 69.34 // p11.2	up
11716386_a_at	RAB11FIP5	26056	chr2:73300509-73340146 (-) // 96.21 // p13.2	up

11723367_a_at	RAB26	25837	chr16:2198624-2204140 (+) // 94.09 // p13.3	up
11722789_a_at	RAB5A	5868	chr3:19988570-20026667 (+) // 92.92 // p24.3	up
11720505_a_at	RAB6B	51560	chr3:133543078-133614691 (-) // 96.1 // q22.1	up
11716287_at	RABAC1	10567	chr19:42460834-42463540 (-) // 80.21 // q13.2	up
11740140_a_at	RASGRP3	25780	chr2:33738941-33789797 (+) // 95.32 // p22.3	up
11736856_at	RASL11A	387496	chr13:27844463-27847827 (+) // 96.82 // q12.2	up
11734527_a_at	RECQL5	9400	chr17:73646441-73663269 (-) // 87.89 // q25.1	up
11747131_at	REG4	83998	chr1:120344945-120354203 (-) // 85.5 // p12	up
11761420_a_at	RGS11	8786	chr16:322258-325937 (-) // 95.42 // p13.3	up
11742143_s_at	RHCE /// RHD	6006 /// 6007	chr1:25598952-25655519 (+) // 82.27 // p36.11 /// chr1:25688740-25747363 (-) // 98.47 // p36.11	up
11734359_a_at	RNASEL	6041	chr1:182542771-182558345 (-) // 76.07 // q25.3	up
11757491_x_at	ROMO1	140823	chr20:34287357-34288899 (+) // 73.56 // q11.22	up
11743266_a_at	RPL34	6164	chr4:109541748-109551640 (+) // 70.6 // q25	up
11748854_a_at	RPUSD4	84881	chr11:126072693-126075676 (-) // 88.95 // q24.2	up
11756025_a_at	S100A13	6284	chr1:153591284-153599673 (-) // 98.09 // q21.3	up
11733442_x_at	SAA1	6288	chr11:18287771-18291514 (+) // 83.42 // p15.1	up
11756072_s_at	SAA1 /// SAA2	6288 /// 6289	chr11:18288455-18290866 (+) // 100.0 // p15.1 /// chr11:18267159-18269537 (-) // 99.48 // p15.1	up
11737184_at	SAA2	6289	chr11:18260475-18269566 (-) // 44.95 // p15.1	up
11729749_at	SAA4	6291	chr11:18252901-18258453 (-) // 78.43 // p15.1	up
11756872_a_at	SAMD4A	23034	chr14:55241679-55255846 (+) // 98.75 // q22.2	up
11718932_a_at	SDCBP2	27111	chr20:1290556-1309883 (-) // 99.68 // p13	up
11725037_a_at	SEC23IP	11196	chr10:121652224-121701833 (+) // 95.15 // q26.11	up
11732334_s_at	SEC31A	22872	chr4:83739467-83812400 (-) // 98.93 // q21.22	up
11720331_at	SELM	140606	chr22:31500758-31503874 (-) // 58.0 // q12.2	up
11715366_a_at	SEPT2	4735	chr2:242255560-242293442 (+) //	up

			85.3 // q37.3	
11720306_at	SERP1	27230	chr3:150259779-150264712 (-) // 98.88 // q25.1	up
11743435_a_at	SEZ6L2	26470	chr16:29882481-29910580 (-) // 98.57 // p11.2	up
11748133_s_at	SLC2A11	66035	chr22:24199137-24226298 (+) // 93.75 // q11.23	up
11740957_x_at	SLC2A6	11182	chr9:136336216-136344276 (-) // 86.18 // q34.2	up
11739934_a_at	SLC39A12	221074	chr10:18240643-18332218 (+) // 97.96 // p12.33	up
11751174_a_at	SLC39A9	55334	chr14:69865102-69925795 (+) // 99.93 // q24.1	up
11748046_a_at	SLC44A4	80736	chr6:31831230-31845448 (-) // 97.01 // p21.33	up
11719222_at	SPINK1	6690	chr5:147204145-147211331 (-) // 70.99 // q32	up
11724215_at	SPRYD4	283377	chr12:56862270-56864769 (+) // 82.47 // q13.3	up
11754274_a_at	ST6GALNAC4	27090	chr9:130670085-130679296 (-) // 98.56 // q34.11	up
11718935_x_at	STK19	8859	chr6:31938942-31949222 (+) // 98.25 // p21.33	up
11754293_a_at	STK40	83931	chr1:36805228-36811194 (-) // 90.61 // p34.3	up
11742479_s_at	SULT1C4	27233	chr2:108994420-109004270 (+) // 98.86 // q12.3	up
11755671_a_at	SYTL2	54843	chr11:85405768-85435756 (-) // 99.79 // q14.1	up
11752602_a_at	TBC1D17	79735	chr19:50380980-50391881 (+) // 99.78 // q13.33	up
11718053_a_at	TBC1D23	55773	chr3:99979660-100044095 (+) // 96.18 // q12.1	up
11718506_at	TFF3	7033	chr21:43731776-43735761 (-) // 99.46 // q22.3	up
11731183_a_at	TFPI	7035	chr2:188343307-188419219 (-) // 95.66 // q32.1	up
11748279_s_at	TM7SF2	7108	chr11:64879349-64883856 (+) // 99.76 // q13.1	up
11722310_a_at	TMC4	147798	chr19:54663845-54676944 (-) // 91.04 // q13.42	up
11716356_at	TMEM167A	153339	chr5:82348671-82373315 (-) // 89.99 // q14.2	up
11732818_at	TMEM171	134285	chr5:72416118-72427641 (+) // 94.05 // q13.2	up
11742824_a_at	TMEM199	147007	chr17:26684603-26689881 (+) // 86.83 // q11.2	up
11732105_at	TMEM208	29100	chr16:67261013-67263181 (+) // 94.64 // q22.1	up
11728022_a_at	TMEM45A	55076	chr3:100211462-100296290 (+) // 96.61 // q12.2	up
11730319_at	TNFRSF11B	4982	chr8:119935800-119964439 (-) // 97.84 // q24.12	up

11743730_at	TNFSF10	8743	chr3:172223302-172241297 (-) // 83.68 // q26.31	up
11718610_at	TP53INP1	94241	chr8:95938199-95961726 (-) // 92.51 // q22.1	up
11715924_a_at	TRAM1	23471	chr8:71485454-71520714 (-) // 92.64 // q13.3	up
11720032_a_at	TRIM29	23650	chr11:119981994-120056148 (-) // 98.13 // q23.3	up
11759923_at	TRIM4	89122	chr7:99500840-99517116 (-) // 35.5 // q22.1	up
11732603_a_at	TRIM45	80263	chr1:117653683-117664476 (-) // 90.94 // p13.1	up
11744301_a_at	TTC17	55761	chr11:43380434-43516470 (+) // 98.79 // p12	up
11744308_a_at	TTC39A	22996	chr1:51752781-51787938 (-) // 97.9 // p32.3	up
11725193_at	TTPAL	79183	chr20:43104531-43123244 (+) // 83.7 // q13.12	up
11727362_x_at	TUBA1A	7846	chr12:49663246-49666967 (+) // 72.38 // q13.12 /// chr12:49578573-49582887 (-) // 97.2 // q13.12 /// chr12:49521740-49523506 (-) // 75.19 // q13.12	up
11718348_a_at	TUSC2	11334	chr3:50362339-50365647 (-) // 95.24 // p21.31	up
11732156_a_at	UFM1	51569	chr13:38923941-38937140 (+) // 93.88 // q13.3	up
11729820_at	USMG5	84833	chr10:105148806-105155780 (-) // 80.55 // q24.33	up
11726826_s_at	VPS37D	155382	chr7:73082157-73086440 (+) // 82.07 // q11.23	up
11727186_a_at	WNT5A	7474	chr3:55499742-55521670 (-) // 96.72 // p14.3	up
11733464_at	XDH	7498	chr2:31557186-31637611 (-) // 91.57 // p23.1	up
11732159_a_at	YIF1A	10897	chr11:66052050-66056626 (-) // 77.52 // q13.2	up
11737803_a_at	YIPF2	78992	chr19:11033445-11039360 (-) // 94.37 // p13.2	up
11724635_at	YOD1	55432	chr1:207217193-207224553 (-) // 98.23 // q32.2	up
11754869_s_at	ZNF267	10308	chr16:31885118-31928622 (+) // 99.61 // p11.2	up
11724634_s_at	ABCB10	23456	chr1:229652328-229694640 (-) // 97.53 // q42.13	down
11727020_at	ABI2	10152	chr2:204192993-204296891 (+) // 93.04 // q33.2	down
11739261_s_at	ACSL3	2181	chr2:223725659-223808118 (+) // 97.83 // q36.1	down
11723946_a_at	ACSL5	51703	chr10:114135862-114188138 (+) // 99.25 // q25.2	down
11722352_s_at	ACTA2	59	chr10:90694832-90712529 (-) //	down

			59.19 // q23.31	
11718016_a_at	ADARB1	104	chr21:46494513-46646475 (+) // 96.44 // q22.3	down
11724877_a_at	AEBP2	121536	chr12:19592425-19675324 (+) // 84.73 // p12.3	down
11723781_at	AGPS	8540	chr2:178257371-178408562 (+) // 96.68 // q31.2	down
11730253_a_at	AHCTF1	25909	chr1:247002399-247094665 (-) // 99.34 // q44 /// chr2:162355653-162364252 (-) // 96.35 // q24.2	down
11725501_at	AHNAK	79026	chr11:62201017-62314332 (-) // 94.06 // q12.3	down
11733030_s_at	AIDA	64853	chr1:222841356-222885864 (-) // 95.98 // q41 /// chr1:78275454-78277620 (-) // 68.72 // p31.1	down
11722671_at	AKAP11	11215	chr13:42846287-42897399 (+) // 96.55 // q14.11	down
11735363_a_at	AKTIP	64400	chr5:24170298-24172386 (+) // 91.32 // p14.2 /// chr16:53525191-53537170 (-) // 98.97 // q12.2	down
11716183_a_at	ALCAM	214	chr3:105085712-105295746 (+) // 96.66 // q13.11	down
11721796_at	ALDH9A1	223	chr1:165631452-165668180 (-) // 92.26 // q24.1	down
11743909_at	ALG14	199857	chr1:95447797-95538507 (-) // 85.06 // p21.3	down
11727577_a_at	ALS2CR4	65062	chr2:202484910-202507686 (-) // 72.55 // q33.1	down
11719004_at	ANKH	56172	chr5:14704912-14871894 (-) // 91.05 // p15.2	down
11744083_at	ANKIB1	54467	chr7:91875496-92030697 (+) // 99.09 // q21.2	down
11717691_a_at	ANKRD13A	88455	chr12:110437194-110477237 (+) // 97.92 // q24.11	down
11719904_s_at	ANKRD40	91369	chr17:48770555-48785270 (-) // 81.31 // q21.33	down
11736047_a_at	ANTXR2	118429	chr4:80822772-80994477 (-) // 89.23 // q21.21	down
11730428_a_at	AP1B1	162	chr22:29723670-29784572 (-) // 98.62 // q12.2	down
11746337_a_at	AP1S2	8905	chr17:58177791-58180146 (-) // 73.6 // q23.1 /// chrX:15849320-15873100 (-) // 89.55 // p22.2	down
11716261_a_at	AP2B1	163	chr17:33913917-34053436 (+) // 94.02 // q12	down
11756982_x_at	AP3S2	10239	chr15:90377561-90437224 (-) // 79.11 // q26.1	down
11742050_a_at	API5	8539	chr11:43333504-43366080 (+) // 97.68 // p12 ///	down

			chr2:178859543-178863598 (-) // 88.63 // q31.2 /// chrX:115235618-115239750 (-) // 93.17 // q23	
11741256_s_at	APOBEC3F	200316	chr22:39436672-39451973 (+) // 43.37 // q13.1	down
11732243_at	APOL6	80830	chr22:36044416-36064407 (+) // 18.3 // q12.3	down
11717791_a_at	APPL2	55198	chr12:105567074-105630010 (-) // 94.15 // q23.3	down
11730288_at	ARHGAP5	394	chr14:32546494-32628932 (+) // 98.56 // q12	down
11747512_s_at	ARHGDIA	396	chr17:79826597-79827833 (-) // 86.17 // q25.3	down
11717697_at	ARIH1	25820	chr15:72766666-72879692 (+) // 95.31 // q24.1	down
11732331_s_at	ARL4C	10123	chr2:235401687-235405693 (-) // 93.35 // q37.1	down
11720466_a_at	ARL6IP1	23204	chr16:18802988-18812871 (-) // 95.48 // p12.3	down
11721254_s_at	ARMCX3	51566	chrX:100877786-100882833 (+) // 76.42 // q22.1	down
11735058_a_at	ARNT2	9915	chr15:80696691-80890269 (+) // 97.98 // q25.1	down
11718724_at	ARRDC3	57561	chr5:90664540-90679176 (-) // 96.24 // q14.3	down
11719761_at	ARRDC4	91947	chr15:98503925-98517063 (+) // 98.54 // q26.3	down
11748793_a_at	ASNS	440	chr7:97481515-97501764 (-) // 99.51 // q21.3	down
11739367_at	ASPH	444	chr8:62413114-62627199 (-) // 97.56 // q12.3	down
11742832_a_at	ASPM	259266	chr1:197053259-197115824 (-) // 97.69 // q31.3	down
11719157_x_at	ASRGL1	80150	chr11:62104773-62160884 (+) // 93.03 // q12.3	down
11725722_at	ATAD2	29028	chr8:124332089-124408705 (-) // 96.45 // q24.13	down
11758854_at	ATL3	25923	chr11:63391553-63439385 (-) // 84.44 // q12.3	down
11739489_a_at	ATP10D	57205	chr4:47487409-47595503 (+) // 96.22 // p12	down
11743863_a_at	ATP2B1	490	chr12:89981825-90049887 (-) // 95.6 // q21.33	down
11728554_at	ATP6V1E2	90423	chr2:46738985-46747176 (-) // 68.68 // p21	down
11720573_at	ATP8B1	5205	chr18:55313675-55399039 (-) // 90.46 // q21.31	down
11726362_a_at	ATXN3	4287	chr14:92524711-92563182 (-) // 72.91 // q32.12	down
11754286_a_at	BCL7B	9275	chr11:93212995-93214785 (-) // 35.92 // q21 /// chr7:72950685-72954275 (-) //	down

			43.67 // q11.23	
11740143_s_at	BEND6	221336	chr6:56819772-56892133 (+) // 98.43 // p12.1	down
11747720_a_at	BIRC5	332	chr17:76210323-76220336 (+) // 96.87 // q25.3	down
11716289_a_at	BLOC1S1	2647	chr12:56109819-56113487 (+) // 66.16 // q13.2	down
11759213_at	BMP8B	656	chr1:40222854-40254533 (-) // 83.91 // p34.2	down
11728699_at	BMPR2	659	chr2:203240944-203432467 (+) // 89.64 // q33.1	down
11726529_s_at	BRCC3	79184	chr5:175735057-175736566 (+) // 30.45 // q35.2 /// chrX:154299694-154350025 (+) // 33.6 // q28	down
11728438_a_at	BRWD1	54014	chr21:40557818-40685556 (-) // 98.43 // q22.2	down
11718056_a_at	BTBD3	22903	chr20:11898012-11907242 (+) // 98.37 // p12.2	down
11726307_at	C10orf84	63877	chr10:120068571-120101783 (-) // 87.03 // q26.11	down
11758960_at	C11orf30	56946	chr11:76155966-76264109 (+) // 90.27 // q13.5	down
11718671_a_at	C11orf49	79096	chr11:46958117-47183798 (+) // 93.04 // p11.2	down
11757934_at	C12orf35	55196	chr12:32145679-32146041 (+) // 99.72 // p11.21	down
11740887_a_at	C13orf23	80209	chr13:39584002-39612252 (-) // 99.55 // q13.3	down
11718413_a_at	C14orf166	51637	chr14:52456192-52471414 (+) // 88.31 // q22.1	down
11726885_s_at	C15orf41	84529	chr15:36936477-37102449 (+) // 91.86 // q14	down
11726209_a_at	C17orf76	388341	chr17:16344924-16395524 (-) // 90.67 // p11.2	down
11729886_at	C18orf54	162681	chr18:51885170-51908404 (+) // 98.04 // q21.2	down
11744918_a_at	C19orf12	83636	chr19:30192074-30206170 (-) // 63.8 // q12	down
11720143_at	C1orf96	126731	chr1:229456758-229478745 (-) // 90.15 // q42.13	down
11725804_a_at	C21orf91	54149	chr21:19161290-19191703 (-) // 97.48 // q21.1	down
11722046_at	C2orf43	60526	chr2:20884577-21022883 (-) // 97.76 // p24.1	down
11728857_at	C3orf34	84984	chr3:196433148-196439159 (-) // 82.36 // q29	down
11741631_a_at	C3orf58	205428	chr3:143691922-143711209 (+) // 96.04 // q24	down
11737948_a_at	C5	727	chr9:123714615-123812549 (-) // 99.54 // q33.2	down
11727287_at	C5orf34	375444	chr5:43486807-43515273 (-) // 96.73 // p12	down

11718824_at	C5orf53	492311	chr5:139505159-139508977 (+) // 84.97 // q31.3	down
11729201_at	C6orf120	387263	chr6:170102215-170106454 (+) // 98.02 // q27	down
11715978_a_at	C6orf48	50854	chr6:31802384-31807541 (+) // 92.69 // p21.33	down
11724103_x_at	C8orf44 /// SGK3	23678 /// 56260	chr8:67624652-67774257 (+) // 83.03 // q13.1	down
11718674_a_at	C9orf5	23731	chr9:111777431-111882230 (-) // 82.14 // q31.3	down
11721829_at	C9orf78	51759	chr9:132589402-132597580 (-) // 82.74 // q34.11	down
11751620_a_at	C9orf86	55684	chr9:139702394-139726891 (+) // 92.93 // q34.3	down
11733708_a_at	CADPS	8618	chr3:62384022-62861064 (-) // 96.66 // p14.2	down
11722530_a_at	CALD1	800	chr7:134464170-134655474 (+) // 95.19 // q33	down
11724759_s_at	CALM1	801	chr14:90863372-90874612 (+) // 97.04 // q32.11	down
11720416_a_at	CAMSAP1	157922	chr9:138700332-138799005 (-) // 99.31 // q34.3	down
11748404_s_at	CAMSAP1L1	23271	chr1:200708906-200829829 (+) // 98.88 // q32.1	down
11738895_at	CAPN10	11132	chr2:241526151-241557122 (+) // 32.06 // q37.3	down
11759328_at	CASC5	57082	chr15:40886217-40956537 (+) // 96.86 // q15.1	down
11720035_s_at	CASP2	835	chr7:142985307-143004789 (+) // 72.07 // q34	down
11722585_a_at	CAST	831	chr5:95997940-96110383 (+) // 97.41 // q15	down
11726010_at	CBR4	84869	chr4:169908740-169931468 (-) // 93.31 // q32.3	down
11754967_s_at	CBX3	11335	chr7:26241368-26252974 (+) // 86.88 // p15.2	down
11716276_a_at	CBX5	23468	chr12:54624727-54653329 (-) // 86.67 // q13.13	down
11758793_at	CCDC109B	55013	chr4:110481353-110609882 (+) // 96.28 // q25	down
11718298_a_at	CCDC25	55246	chr8:27590834-27630170 (-) // 98.72 // p21.1	down
11719949_a_at	CCDC99	54908	chr5:169010361-169031782 (+) // 71.88 // q35.1	down
11723939_a_at	CCNB1	891	chr5:68462836-68474067 (+) // 90.74 // q13.2	down
11723930_at	CCT5	22948	chr5:10250281-10267258 (+) // 89.37 // p15.2	down
11737913_s_at	CCT7	10574	chr2:73461404-73480147 (+) // 98.99 // p13.2	down
11735329_a_at	CD47	961	chr3:107761938-107809935 (-) // 94.48 // q13.12	down
11718100_s_at	CD59	966	chr11:33724555-33744276 (-) //	down

			84.95 // p13	
11759740_x_at	CDC23	8697	chr5:137541560-137549033 (-) // 84.99 // q31.2	down
11743402_at	CDC27	996	chr17:45195068-45266663 (-) // 94.09 // q21.32	down
11741540_a_at	CDC42BPA	8476	chr1:227177566-227505826 (-) // 96.34 // q42.13	down
11732057_a_at	CDC7	8317	chr1:91966403-91991321 (+) // 93.73 // p22.2	down
11758478_s_at	CDCA7	83879	chr2:174233163-174233630 (+) // 99.79 // q31.1	down
11729579_at	CDH13	1012	chr16:82660568-83830201 (+) // 91.69 // q23.3	down
11745843_a_at	CDK1	983	chr10:62538247-62555217 (+) // 85.06 // q21.2	down
11720622_a_at	CDK16	5127	chrX:47078075-47089396 (+) // 93.74 // p11.23	down
11741208_a_at	CDK2	1017	chr12:56360555-56366567 (+) // 97.44 // q13.2	down
11753378_a_at	CDK4	1019	chr12:58142302-58146109 (-) // 100.0 // q14.1	down
11763387_s_at	CDK8	1024	chr13:26978833-26979372 (+) // 98.18 // q12.13	down
11758661_s_at	CDKN2C	1031	chr1:51440006-51440305 (+) // 100.0 // p32.3	down
11753880_x_at	CDKN3	1033	chr14:54866610-54886834 (+) // 95.98 // q22.2	down
11719642_at	CDS1	1040	chr4:85504056-85572487 (+) // 97.44 // q21.23	down
11717693_at	CEBPG	1054	chr19:33864235-33873592 (+) // 95.15 // q13.11	down
11743296_a_at	CENPF	1063	chr1:214776527-214837920 (+) // 99.15 // q41	down
11739328_x_at	CENPH	64946	chr5:68485374-68506183 (+) // 76.3 // q13.2	down
11727973_a_at	CENPN	55839	chr16:81039517-81065088 (+) // 67.13 // q23.2	down
11720247_a_at	CEP55	55165	chr10:95256356-95288851 (+) // 95.49 // q23.33	down
11762596_a_at	CEP57L1	285753	chr10:72149121-72149922 (+) // 40.09 // q22.1 /// chr6:109416360-109477999 (+) // 80.69 // q21	down
11757453_s_at	CEP68	23177	chr2:65313535-65314137 (+) // 99.18 // p14	down
11728361_a_at	CHCHD7	79145	chr8:57124314-57131357 (+) // 83.29 // q12.1	down
11723354_s_at	CHD7	55636	chr8:61591336-61779749 (+) // 98.85 // q12.1	down
11754262_s_at	CHD9	80205	chr16:53357509-53361408 (+) // 97.33 // q12.2	down
11732736_at	CHSY3	337876	chr5:129240164-129522326 (+) // 95.85 // q23.3	down

11727821_s_at	CISD1	55847	chr10:60028884-60049563 (+) // 93.68 // q21.1	down
11755868_x_at	CLCC1	23155	chr1:109472560-109506072 (-) // 73.61 // p13.3	down
11729621_at	CLCN4	1183	chrX:10124872-10205705 (+) // 94.17 // p22.2	down
11719163_a_at	CLCN5	1184	chrX:49687224-49863887 (+) // 92.28 // p11.23	down
11754405_s_at	CLIC4	25932	chr1:25170191-25170766 (+) // 91.55 // p36.11 /// chr5:180136520-180137100 (-) // 89.47 // q35.3	down
11722507_a_at	CLIC5	53405	chr6:45866187-46048085 (-) // 98.84 // p21.1	down
11754044_at	CLSPN	63967	chr1:36200238-36201625 (-) // 66.98 // p34.3	down
11756353_a_at	CLVS1	157807	chr8:62200533-62414428 (+) // 99.71 // q12.3	down
11738807_s_at	CMIP	80790	chr16:81528953-81745365 (+) // 94.24 // q23.2	down
11727420_at	CNBP	7555	chr3:128886658-128902823 (-) // 71.45 // q21.3	down
11722929_a_at	CNKSR3	154043	chr6:154726293-154831803 (-) // 99.77 // q25.2	down
11743297_a_at	CNOT6	57472	chr5:179921403-180005404 (+) // 97.37 // q35.3	down
11718583_a_at	CNPY2	10330	chr12:56703974-56710132 (-) // 99.59 // q13.3	down
11730533_a_at	COL13A1	1305	chr10:71561590-71718900 (+) // 97.81 // q22.1	down
11753590_a_at	COMMD9	29099	chr11:36296181-36310962 (-) // 99.83 // p13	down
11743141_a_at	COX11	1353	chr6:28414702-28417105 (+) // 82.07 // p22.1 /// chr17:53029266-53046059 (-) // 95.18 // q22	down
11718556_s_at	CREB3L2	64764	chr7:137559724-137686847 (-) // 96.8 // q33	down
11723280_at	CREBZF	58487	chr11:85368395-85376185 (-) // 93.51 // q14.1	down
11733353_at	CRTAM	56253	chr11:122709207-122743347 (+) // 85.06 // q24.1	down
11746367_a_at	CSE1L	1434	chr20:47662785-47711357 (+) // 89.2 // q13.13	down
11740002_a_at	CTBP2	1488	chr5:97912042-97914160 (+) // 57.96 // q15 /// chr1:68625772-68629239 (-) // 92.67 // p31.3 /// chr10:126676417-126849141 (-) // 98.2 // q26.13	down
11731575_a_at	CTBS	1486	chr1:85018803-85040163 (-) // 89.17 // p22.3	down
11757500_a_at	CTDSPL	10217	chr3:38025592-38025959 (+) //	down

			91.07 // p22.2	
11715441_a_at	CTGF	1490	chr6:132268798-132272580 (-) // 94.17 // q23.2	down
11720494_a_at	CTNNAL1	8727	chr9:111704848-111775874 (-) // 98.89 // q31.3	down
11761290_x_at	CTU2 /// PRKAR2A	348180 /// 5576	chr16:88772910-88779212 (+) // 54.28 // q24.3	down
11720095_a_at	CUL4B	8450	chrX:119658466-119694936 (-) // 98.2 // q24	down
11723077_at	CXorf26	51260	chrX:75392741-75398424 (+) // 87.99 // q13.3	down
11720411_s_at	CYP1B1	1545	chr2:38294650-38303323 (-) // 99.15 // p22.2	down
11734902_a_at	CYP4V2	285440	chr4:187112470-187134612 (+) // 83.43 // q35.2	down
11726670_a_at	DCLRE1B	64858	chr1:114447767-114456708 (+) // 86.37 // p13.2	down
11729488_x_at	DCLRE1C	64421	chr10:14947927-14996094 (-) // 59.05 // p13	down
11719471_at	DCP1A	55802	chr3:53317442-53381654 (-) // 94.34 // p21.1	down
11759818_a_at	DDHD2	23259	chr8:38089470-38120282 (+) // 99.34 // p11.23	down
11730111_a_at	DEPDC1	55635	chr1:68939834-68962799 (-) // 78.37 // p31.2	down
11742239_a_at	DEPDC1B	55789	chr5:59892739-59995993 (-) // 94.68 // q12.1	down
11718998_x_at	DHFR	1719	chr18:23747811-23751321 (-) // 38.53 // q11.2 /// chr5:79922190-79950802 (-) // 44.53 // q14.1	down
11745415_a_at	DIAPH1	1729	chr5:140894588-140998496 (-) // 98.16 // q31.3	down
11744730_s_at	DLEU2 /// DLEU2L	79469 /// 8847	chr13:50617572-50656177 (-) // 96.36 // q14.2	down
11757609_s_at	DLG5	9231	chrX:72075054-72075620 (+) // 89.75 // q13.2 /// chr10:79550548-79551114 (-) // 100.0 // q22.3 /// chrX:72084903-72085469 (-) // 89.75 // q13.2	down
11720932_s_at	DLST	1743	chr14:75348595-75370443 (+) // 98.64 // q24.3 /// chr1:76207710-76210467 (-) // 92.51 // p31.1	down
11740075_at	DMGDH	29958	chr5:78293386-78365455 (-) // 99.97 // q14.1	down
11755657_s_at	DMXL1	1657	chr5:118572008-118584815 (+) // 86.1 // q23.1	down
11723648_at	DNAJA2	10294	chr16:46989273-47007699 (-) // 97.15 // q11.2	down
11762410_a_at	DNAJB14	79982	chr15:66062041-66064346 (-) // 64.56 // q22.31 ///	down

			chr4:100819916-100851691 (-) // 97.86 // q23	
11743537_a_at	DNAJC18	202052	chr5:138747380-138780171 (-) // 86.77 // q31.2	down
11720773_x_at	DNAJC19	131118	chr14:45759135-45759676 (+) // 35.2 // q21.2 /// chr3:180701496-180707561 (-) // 84.42 // q26.33	down
11736405_a_at	DNMT1	1786	chr19:10244022-10305811 (-) // 99.25 // p13.2	down
11717200_a_at	DPM2	8818	chr9:130697377-130701204 (-) // 77.67 // q34.11	down
11716723_at	DPY19L1	23333	chr7:32852368-32885666 (+) // 69.23 // p14.3 /// chr7:34968492-35077653 (-) // 97.68 // p14.3	down
11715751_a_at	DPYSL3	1809	chr5:146770370-146833716 (-) // 97.36 // q32	down
11721158_a_at	DRAM2	128338	chr1:111659954-111682838 (-) // 97.74 // p13.3	down
11741980_a_at	DUSP6	1848	chr12:89741838-89746296 (-) // 98.67 // q21.33	down
11726597_a_at	DUT	1854	chr15:48623622-48635570 (+) // 90.36 // q21.1	down
11747943_a_at	E2F8	79733	chr11:19245879-19263204 (-) // 97.67 // p15.1	down
11724301_a_at	EEA1	8411	chr12:93166020-93323107 (-) // 98.46 // q22	down
11728379_at	EFCAB2	84288	chr1:245133040-245251139 (+) // 95.99 // q44	down
11725642_at	EFHD2	79180	chr1:15736391-15756829 (+) // 98.0 // p36.21	down
11725102_at	EGFR	1956	chr7:55086713-55275325 (+) // 94.55 // p11.2	down
11733856_s_at	EGLN1	54583	chr1:231499496-231560790 (-) // 85.4 // q42.2	down
11754334_s_at	EGR1	1958	chr5:137804475-137804992 (+) // 81.29 // q31.2	down
11722728_a_at	EGR2	1959	chr10:64571756-64578927 (-) // 99.41 // q21.3	down
11746045_s_at	ELF2	1998	chr2:186410878-186412545 (-) // 50.23 // q32.1 /// chr4:139979431-140005613 (-) // 94.61 // q31.1	down
11721574_a_at	ELF4	2000	chrX:129198458-129244473 (-) // 94.1 // q26.1	down
11716654_a_at	EML4	27436	chr2:42396492-42560624 (+) // 98.36 // p21	down
11718098_at	ENDOD1	23052	chr11:94822973-94865809 (+) // 92.24 // q21	down
11743265_a_at	ENSA	2029	chr1:150595360-150602098 (-) // 90.88 // q21.3	down
11715411_a_at	EPAS1	2034	chr2:46524540-46613836 (+) //	down

			96.03 // p21	
11721168_a_at	EPB41L1	2036	chr20:34742661-34820719 (+) // 93.83 // q11.23	down
11717470_at	EPT1	85465	chr2:26568953-26618753 (+) // 93.8 // p23.3	down
11721043_at	ERI1	90459	chr8:8859692-8890848 (+) // 83.65 // p23.1	down
11730914_a_at	ERLIN1	10613	chr10:101909846-101945814 (-) // 97.67 // q24.31	down
11727968_at	ESCO2	157570	chr8:27631902-27662802 (+) // 84.48 // p21.1	down
11720352_at	ETV5	2119	chr3:185764102-185826901 (-) // 92.57 // q27.2	down
11759905_a_at	EXD3	54932	chr9:140260597-140317611 (-) // 96.6 // q34.3	down
11719458_at	EXOC5	10640	chr14:57668879-57735726 (-) // 90.51 // q22.3	down
11717223_a_at	EXTL3	2137	chr8:28457985-28611204 (+) // 96.83 // p21.1	down
11717657_a_at	EZH2	2146	chr7:148504475-148581411 (-) // 95.73 // q36.1	down
11754939_x_at	FADS2	9415	chr11:61623751-61634819 (+) // 90.87 // q12.2	down
11716002_at	FAF2	23197	chr5:175875374-175937531 (+) // 94.47 // q35.2	down
11722128_at	FAM102B	284611	chr1:109102970-109181949 (+) // 94.44 // p13.3	down
11752456_a_at	FAM108C1	58489	chr15:80987651-81047678 (+) // 88.04 // q25.1	down
11739933_a_at	FAM111B	374393	chr11:58874657-58894312 (+) // 66.83 // q12.1	down
11750692_s_at	FAM115A	9747	chr7:143295130-143305200 (+) // 92.33 // q35 /// chr7:143550920-143582364 (-) // 98.69 // q35	down
11719737_a_at	FAM134B	54463	chr5:16473146-16617210 (-) // 93.2 // p15.1	down
11755906_a_at	FAM135A	57579	chr6:71238010-71270875 (+) // 93.67 // q13	down
11754677_a_at	FAM169A	26049	chr5:74073425-74191788 (-) // 95.41 // q13.3	down
11719362_at	FAM178A	55719	chr10:102672325-102724889 (+) // 98.28 // q24.31	down
11755408_a_at	FAM199X	139231	chrX:103430798-103436125 (+) // 87.14 // q22.2	down
11752768_s_at	FAM54B	56181	chr1:26155996-26158921 (+) // 53.15 // p36.11 /// chr18:31523028-31603560 (-) // 46.72 // q12.1	down
11723747_a_at	FAM64A	54478	chr17:6347758-6354780 (+) // 95.58 // p13.2	down
11740517_a_at	FAM76A	199870	chr1:28052489-28089421 (+) // 85.28 // p35.3	down

11739255_x_at	FAM84B	157638	chr8:127564682-127570706 (-) // 94.05 // q24.21	down
11719895_x_at	FAM91A1	157769	chr1:149261011-149266468 (+) // 90.23 // q21.2 /// chr8:124780678-124827688 (+) // 98.14 // q24.13	down
11719894_s_at	FAM91A1 /// FAM91A2	157769 /// 57234	chr1:149261011-149266468 (+) // 90.23 // q21.2 /// chr8:124780678-124827688 (+) // 98.14 // q24.13	down
11758994_at	FAR2	55711	chr12:29376555-29488548 (+) // 98.94 // p11.22	down
11750165_a_at	FARSB	10056	chr2:223436486-223520808 (-) // 98.32 // q36.1	down
11731124_at	FBXO36	130888	chr17:66091209-66092631 (+) // 38.81 // q24.2 /// chr2:230787206-230877825 (+) // 90.23 // q36.3	down
11724963_s_at	FBXO5	26271	chr6:153291669-153304753 (-) // 97.24 // q25.2	down
11726997_a_at	FIBP	9158	chr11:65651210-65656010 (-) // 96.69 // q13.1	down
11715425_x_at	FLNA	2316	chrX:153576899-153599744 (-) // 96.24 // q28	down
11759342_at	FMN1	342184	chr15:33057746-33360085 (-) // 96.18 // q13.3	down
11737831_a_at	FNBP1L	54874	chr1:93965023-94020216 (+) // 98.62 // p22.1	down
11755772_a_at	FOXO1	2308	chr13:41129896-41241189 (-) // 98.09 // q14.11	down
11730808_at	FPGT	8790	chr1:74663921-74673698 (+) // 83.42 // p31.1	down
11736507_s_at	FRMD6	122786	chr14:52118574-52197445 (+) // 93.34 // q22.1	down
11725141_a_at	FUBP3	8939	chr9:133454947-133513739 (+) // 97.62 // q34.11	down
11757005_a_at	FUCA1	2517	chr1:24175298-24194780 (-) // 100.0 // p36.11	down
11760826_at	GALNT3	2591	chr2:166626165-166650706 (-) // 92.07 // q24.3	down
11722408_at	GALNT7	51809	chr4:174089912-174245118 (+) // 97.45 // q34.1	down
11719353_s_at	GCC2	9648	chr2:109065576-109125853 (+) // 95.67 // q12.3	down
11758456_s_at	GFPT1	2673	chr2:69548817-69549293 (-) // 99.79 // p13.3	down
11728863_at	GINS1	9837	chr20:25388320-25427956 (+) // 42.53 // p11.21	down
11732922_at	GJA3	2700	chr13:20712396-20735183 (-) // 95.98 // q12.11	down
11722307_at	GLCE	26035	chr15:69452972-69564546 (+) // 95.59 // q23	down
11739882_a_at	GLIS3	169792	chr9:3824127-4300035 (-) //	down

			92.37 // p24.2	
11746439_a_at	GLO1	2739	chr6:38643739-38670917 (-) // 90.59 // p21.2	down
11737747_a_at	GMFB	2764	chr14:54941208-54955815 (-) // 99.15 // q22.2	down
11727848_a_at	GNA12	2768	chr7:2767742-2883962 (-) // 94.26 // p22.3	down
11717681_at	GNA13	10672	chr17:63005406-63052920 (-) // 98.58 // q24.1	down
11721381_at	GNPTAB	79158	chr12:102139274-102224716 (-) // 88.37 // q23.2	down
11716752_x_at	GNS	2799	chr12:65107006-65153227 (-) // 88.0 // q14.3	down
11750722_a_at	GPHN	10243	chr14:66975230-67647914 (+) // 99.92 // q23.3	down
11732319_a_at	GPR1	2825	chr2:207039827-207082771 (-) // 86.05 // q33.3	down
11743011_a_at	GPR137B	7107	chr1:236305709-236372207 (+) // 93.52 // q42.3	down
11739977_at	GPR180	160897	chr13:95253856-95282698 (+) // 94.94 // q32.1	down
11729645_x_at	GPX8	493869	chr5:54455945-54463339 (+) // 83.39 // q11.2	down
11719301_s_at	GRPEL2	134266	chr5:148724375-148734146 (+) // 77.49 // q32	down
11732171_x_at	GRSF1	2926	chr4:71682122-71705607 (-) // 69.33 // q13.3	down
11723520_a_at	GRTP1	79774	chr13:113978327-114018444 (-) // 96.84 // q34	down
11719622_at	GTPBP1	9567	chr22:39101727-39129586 (+) // 86.95 // q13.1	down
11724519_a_at	GUF1	60558	chr4:44680411-44702944 (+) // 68.56 // p12	down
11740663_a_at	GXYLT1	283464	chr12:42475649-42538673 (-) // 88.31 // q12	down
11745819_a_at	H1F0	3005	chr22:38201235-38203439 (+) // 94.97 // q13.1	down
11743694_at	H2AFV	94239	chr7:44866387-44882954 (-) // 65.86 // p13	down
11733539_a_at	HEATR7A	727957	chr8:145202938-145268366 (+) // 80.45 // q24.3	down
11718309_at	HEG1	57493	chr3:124684553-124774802 (-) // 93.14 // q21.2	down
11759141_at	HIP1	3092	chr7:75162618-75368283 (-) // 86.45 // q11.23	down
11719409_a_at	HIPK2	28996	chr7:139246315-139477596 (-) // 94.67 // q34	down
11756138_a_at	HK2	3099	chr2:75062296-75120478 (+) // 98.47 // p12 /// chrX:79824925-79830043 (-) // 91.0 // q21.1	down
11737371_a_at	HLCS	3141	chr21:38123189-38353125 (-) // 96.12 // q22.13	down

11732091_a_at	HMG20A	10363	chr15:77713242-77777943 (+) // 94.6 // q24.3	down
11740656_a_at	HMGA2	8091	chr12:66218239-66360070 (+) // 91.11 // q14.3	down
11754183_s_at	HMGB3	3149	chrX:150151762-150159246 (+) // 87.43 // q28	down
11716987_a_at	HMGCS1	3157	chr5:43289207-43313684 (-) // 94.68 // p12	down
11723197_at	HNRNPA3	220988	chr2:178077022-178088686 (+) // 93.56 // q31.2 /// chr10:44282572-44285890 (-) // 45.75 // q11.21	down
11723199_s_at	HNRNPA3 /// HNRNPA3P1	10151 /// 220988	chr2:178077022-178088686 (+) // 93.56 // q31.2 /// chr10:44282572-44285890 (-) // 45.75 // q11.21	down
11729146_a_at	HNRNPD	3184	chr4:83274213-83295505 (-) // 93.28 // q21.22	down
11758027_s_at	HOOK1	51361	chr1:60341429-60341994 (+) // 98.6 // p32.1	down
11743655_a_at	HPGD	3248	chr4:175411281-175444049 (-) // 97.21 // q34.1	down
11725668_at	HS2ST1	9653	chr1:87380334-87575658 (+) // 90.34 // p22.3	down
11755876_a_at	IFRD2	7866	chr3:50325173-50330349 (-) // 97.41 // p21.31	down
11727320_at	IGFL2	147920	chr19:46651038-46664556 (+) // 84.66 // q13.32	down
11731845_a_at	IGSF3	3321	chr1:117117030-117210377 (-) // 98.49 // p13.1	down
11731944_at	IGSF5	150084	chr21:41137460-41174023 (+) // 86.29 // q22.2	down
11751009_s_at	IK	3550	chr5:140027425-140042063 (+) // 92.11 // q31.3 /// chr2:58687307-58689167 (-) // 85.35 // p16.1	down
11727905_a_at	IL13RA1	3597	chrX:117861257-117928929 (+) // 91.26 // q24	down
11740881_x_at	IL15	3600	chr4:142557748-142654611 (+) // 97.94 // q31.21	down
11723173_at	IL17RD	54756	chr3:57124011-57199403 (-) // 84.1 // p14.3	down
11743858_at	IL33	90865	chr9:6215776-6257982 (+) // 99.19 // p24.1	down
11759037_s_at	IL6ST	3572	chr5:55232192-55290821 (-) // 96.26 // q11.2	down
11726159_a_at	IQCG	84223	chr3:197615945-197686892 (-) // 97.92 // q29	down
11749201_a_at	IQCJ-SCHIP1 /// SCHIP1	100505385 /// 29970	chr3:158787052-159614563 (+) // 96.58 // q25.32	down
11754475_a_at	IQCK	124152	chr16:19727777-19869067 (+) // 96.4 // p12.3	down
11721061_a_at	IQGAP2	10788	chr5:75698825-76004056 (+) //	down

			98.76 // q13.3	
11756521_a_at	IREB2	3658	chr15:78792463-78793794 (+) // 55.31 // q25.1	down
11716468_s_at	ISCA1	81689	chr2:129274849-129276832 (-) // 78.65 // q14.3 /// chr9:88879460-88897676 (-) // 88.73 // q21.33	down
11730523_at	ISM1	140862	chr20:13202446-13281298 (+) // 94.49 // p12.1	down
11719100_at	ITFG3	83986	chr16:284544-316119 (+) // 96.22 // p13.3	down
11737219_a_at	ITGB1	3688	chr10:33189246-33247155 (-) // 91.17 // p11.22	down
11754810_a_at	ITGB5	3693	chr3:124481794-124489066 (-) // 95.68 // q21.2	down
11722503_at	ITGB8	3696	chr7:20370724-20455382 (+) // 90.43 // p21.1	down
11742964_a_at	ITPR3	3710	chr6:33589155-33664351 (+) // 99.38 // p21.31	down
11720825_s_at	JAG1	182	chr20:10618333-10654755 (-) // 96.82 // p12.2	down
11755223_a_at	JHDM1D	80853	chr7:139784655-139813384 (-) // 93.8 // q34	down
11725822_a_at	JPH1	56704	chr8:75146934-75233678 (-) // 98.24 // q21.11	down
11758166_s_at	KAL1	3730	chrY:16031240-16031901 (+) // 87.46 // q11.221	down
11723325_a_at	KCNQ1	3784	chr11:2466220-2870340 (+) // 94.98 // p15.5	down
11758284_s_at	KCTD9	54793	chr11:112055446-112056061 (+) // 93.7 // q23.1 /// chr17:20017849-20018463 (+) // 89.29 // p11.2 /// chr9:37479845-37480442 (+) // 87.09 // p13.2 /// chrX:104652947-104653561 (+) // 95.59 // q22.3 /// chr5:37504443-37505056 (-) // 91.5 // p13.2 /// chr8:25285538-25286148 (-) // 96.06 // p21.2 /// chr8:31075696-31076304 (-) // 90.71 // p12	down
11730180_s_at	KDSR	2531	chr18:60994807-61034516 (-) // 81.73 // q21.33	down
11721807_a_at	KIAA0090	23065	chr1:19541883-19578291 (-) // 85.03 // p36.13	down
11735833_a_at	KIAA1199	57214	chr15:81071683-81243999 (+) // 97.76 // q25.1	down
11737795_a_at	KIAA1524	57650	chr3:108268719-108308491 (-) // 98.9 // q13.13	down
11746232_a_at	KIDINS220	57498	chr2:8869058-8977726 (-) // 98.16 // p25.1	down

11727489_a_at	KIF11	3832	chr10:94352824-94415152 (+) // 91.06 // q23.33	down
11731887_at	KIF14	9928	chr1:200520627-200589862 (-) // 94.85 // q32.1	down
11723010_a_at	KIF20A	10112	chr5:137514404-137523851 (+) // 99.92 // q31.2	down
11721932_a_at	KIF23	9493	chr15:69706687-69740766 (+) // 98.27 // q23	down
11739068_a_at	KIFAP3	22920	chr1:169890471-170043897 (-) // 98.98 // q24.2	down
11728954_a_at	KITLG	4254	chr12:88886569-88974238 (-) // 94.59 // q21.32	down
11733068_s_at	KLF10	7071	chr8:103661011-103668130 (-) // 95.94 // q22.3	down
11727145_s_at	KLF11	8462	chr2:10183681-10194963 (+) // 97.38 // p25.1	down
11717326_at	KLF9	687	chr9:72999512-73029573 (-) // 95.75 // q21.12	down
11760228_s_at	KLHL23	151230	chr2:170590355-170608394 (+) // 69.17 // q31.1	down
11718199_at	KPNA3	3839	chr13:50273442-50367214 (-) // 96.14 // q14.2	down
11723269_at	KPNA6	23633	chr1:32573626-32642164 (+) // 90.45 // p35.1	down
11716051_at	LAMC1	3915	chr1:182992594-183114726 (+) // 97.74 // q25.3	down
11719614_a_at	LARP4	113251	chr12:50794639-50873787 (+) // 97.82 // q13.12	down
11726871_s_at	LEPR	3953	chr1:65886317-66101111 (+) // 99.36 // p31.3	down
11759120_a_at	LGR5	8549	chr12:71833571-71980086 (+) // 99.44 // q21.1	down
11736709_a_at	LIMCH1	22998	chr4:41614918-41702060 (+) // 97.71 // p13	down
11740773_s_at	LIN54	132660	chr4:83845755-83931982 (-) // 85.32 // q21.22	down
11724652_a_at	LMBR1	64327	chr7:156473574-156685902 (-) // 98.39 // q36.3	down
11744604_at	LOC100288413	100288413	chr4:53609691-53617566 (-) // 18.48 // q12	down
11763303_at	LOC100506748	100506748	chr2:114464355-114468151 (-) // 62.62 // q14.1	down
11759610_at	LOC404266	404266	chr17:46667822-46683762 (+) // 81.93 // q21.32	down
11760121_at	LONP2	83752	chr16:48278243-48396896 (+) // 73.82 // q12.1	down
11725417_a_at	LPCAT2	54947	chr16:55542674-55620582 (+) // 92.02 // q12.2	down
11723468_at	LPGAT1	9926	chr1:211916799-212004114 (-) // 88.46 // q32.3	down
11742722_at	LRRC58	116064	chr3:120043318-120068214 (-) // 81.65 // q13.33	down
11752870_a_at	LRRCC1	85444	chr8:86019322-86047151 (+) //	down

			95.74 // q21.2	
11736533_s_at	LRRFIP1	9208	chr2:238536223-238690286 (+) // 94.48 // q37.3	down
11736150_at	LYRM2	57226	chr6:90341944-90348475 (-) // 64.9 // q15	down
11717241_a_at	MAEA	10296	chr4:1283640-1333925 (+) // 98.22 // p16.3	down
11756693_a_at	MAGED1	9500	chrX:51636734-51641253 (+) // 95.0 // p11.22	down
11757043_a_at	MAGED2	10916	chrX:54834795-54841208 (+) // 95.17 // p11.21	down
11723447_at	MALL	7851	chr2:111037181-111045079 (+) // 58.09 // q13 /// chr2:110841450-110874143 (-) // 83.21 // q13	down
11733114_a_at	MANBAL	63905	chr20:35929608-35945661 (+) // 83.26 // q11.23	down
11719499_at	MAOB	4129	chrX:43625859-43741721 (-) // 89.24 // p11.3	down
11724902_a_at	MAP3K14	9020	chr17:43340490-43394511 (-) // 97.72 // q21.31	down
11736006_at	MAPK1IP1L	93487	chr14:55518355-55536912 (+) // 89.37 // q22.3	down
11744024_a_at	MARS	4141	chr12:57881689-57910432 (+) // 90.27 // q13.3	down
11717009_s_at	MCFD2	90411	chr2:47129012-47136317 (-) // 42.22 // p21	down
11743693_a_at	MCM4	4173	chr8:48872762-48891078 (+) // 82.81 // q11.21	down
11722383_a_at	MDM1	56890	chr12:68688345-68726161 (-) // 97.92 // q15	down
11756418_a_at	MEAF6	64769	chr1:37958778-37980417 (-) // 90.0 // p34.3	down
11759175_at	MED28	80306	chr4:17616279-17627249 (+) // 81.76 // p15.32	down
11746193_a_at	METTL3	56339	chr14:21966305-21979482 (-) // 99.51 // q11.2	down
11741010_a_at	MFN2	9927	chr1:12040237-12073571 (+) // 99.78 // p36.22	down
11734534_s_at	MFSD6	54842	chr2:191273080-191367040 (+) // 96.61 // q32.2	down
11750984_a_at	MGAT2	4247	chr14:50087532-50089583 (+) // 100.0 // q21.3	down
11721144_a_at	MKI67	4288	chr10:129894922-129924655 (-) // 98.51 // q26.2	down
11729551_s_at	MKLN1	4289	chr7:130794854-131181398 (+) // 96.35 // q32.3	down
11754396_a_at	MLLT3	4300	chr9:20344803-20414267 (-) // 97.45 // p21.3	down
11733445_at	MMS22L	253714	chr6:97590036-97731052 (-) // 90.35 // q16.1	down
11725167_at	MOBKL2B	79817	chr9:27325208-27529851 (-) // 85.65 // p21.2	down

11717643_a_at	MRPL24	79590	chr1:156707094-156710906 (-) // 67.38 // q23.1	down
11743334_a_at	MRPL35	51318	chr2:86426477-86440917 (+) // 78.57 // p11.2	down
11715790_a_at	MRPL37	51253	chr1:54665820-54684056 (+) // 96.21 // p32.3	down
11725683_at	MRS2	57380	chr6:24403135-24426418 (+) // 97.61 // p22.3	down
11716413_x_at	MT1E	4493	chr16:56659386-56661020 (+) // 78.1 // q12.2	down
11717710_a_at	MTFP1	51537	chr22:30821451-30825040 (+) // 79.23 // q12.2	down
11717711_s_at	MTFP1 /// SEC14L2	23541 /// 51537	chr22:30821451-30825040 (+) // 79.23 // q12.2	down
11728984_a_at	MTHFD1L	25902	chr6:151186690-151423021 (+) // 96.85 // q25.1	down
11762057_at	MTHFD2L	441024	chr4:74979890-75107914 (+) // 57.93 // q13.3	down
11727722_a_at	MYB	4602	chr6:135502449-135540310 (+) // 93.45 // q23.3	down
11728523_a_at	MYCL1	4610	chr1:40361099-40367687 (-) // 97.97 // p34.2	down
11754984_s_at	MYH10	4628	chr17:8377531-8534068 (-) // 99.06 // p13.1	down
11742414_x_at	MYO10	4651	chr5:16662016-16936385 (-) // 85.04 // p15.1	down
11716452_a_at	MYO1C	4641	chr17:1367394-1396106 (-) // 88.85 // p13.3	down
11726456_at	MYO5B	4645	chr18:47349100-47721452 (-) // 99.25 // q21.1	down
11715908_at	MYO6	4646	chr6:76458892-76629254 (+) // 91.39 // q14.1	down
11739508_a_at	NAA30	122830	chr14:57857270-57879699 (+) // 97.26 // q22.3	down
11739475_s_at	NAB1	4664	chr2:191513847-191557492 (+) // 98.45 // q32.2	down
11717852_at	NAGLU	4669	chr17:40687945-40696671 (+) // 88.81 // q21.2	down
11762777_a_at	NAP1L1	4673	chr12:76443055-76478392 (-) // 99.79 // q21.2	down
11715746_a_at	NBL1	4681	chr1:19969722-19984945 (+) // 86.69 // p36.13	down
11722825_at	NCAPG	64151	chr4:17812435-17846629 (+) // 98.45 // p15.31	down
11757197_s_at	NCRNA00201	284702	chr1:245003940-245010243 (-) // 85.17 // q44	down
11756077_a_at	NDE1	54820	chr16:15815493-15818991 (+) // 64.18 // p13.11	down
11730881_a_at	NEBL	10529	chr10:21068904-21186531 (-) // 98.58 // p12.31	down
11740287_a_at	NEDD1	121441	chr12:97301000-97347461 (+) // 97.91 // q23.1	down
11722251_a_at	NEK2	4751	chr1:211836113-211849184 (-) //	down

			96.39 // q32.3	
11718464_s_at	NEURL1B	54492	chr5:172068275-172118543 (+) // 90.9 // q35.1	down
11722661_at	NFE2L3	9603	chr17:45500853-45504058 (+) // 72.74 // q21.32 /// chr18:57636678-57639976 (+) // 82.91 // q21.32 /// chr7:26191857-26226745 (+) // 95.42 // p15.2	down
11758230_s_at	NFIA	4774	chr1:61923229-61923901 (+) // 87.06 // p31.3	down
11755756_a_at	NHSL1	57224	chr6:138743182-138768326 (-) // 97.64 // q23.3	down
11743377_a_at	NIN	51199	chr14:51186483-51297858 (-) // 91.53 // q22.1	down
11759191_at	NIPAL2	79815	chr8:99202053-99306621 (-) // 84.71 // q22.2	down
11725627_at	NIPAL3	57185	chr1:24742239-24799473 (+) // 86.99 // p36.11	down
11715733_a_at	NIPSNAP1	8508	chr22:29950796-29977328 (-) // 89.36 // q12.2	down
11736283_s_at	NKX3-1	4824	chr8:23536205-23540450 (-) // 97.29 // p21.2	down
11718435_a_at	NPNT	255743	chr4:106816604-106892816 (+) // 94.19 // q24	down
11717568_s_at	NQO1	1728	chr16:69743303-69760557 (-) // 84.7 // q22.1	down
11757751_s_at	NRP1	8829	chr10:33466494-33467764 (-) // 95.38 // p11.22	down
11755207_a_at	NT5E	4907	chr6:86159826-86205498 (+) // 92.66 // q14.3	down
11728647_at	NUCKS1	64710	chr1:205681946-205719257 (-) // 94.14 // q32.1	down
11732160_a_at	NUDCD1	84955	chr8:110253147-110346614 (-) // 90.31 // q23.1	down
11722442_a_at	NUF2	83540	chr1:163291689-163325554 (+) // 97.51 // q23.3	down
11744020_at	NUP210	23225	chr3:13357734-13461809 (-) // 97.59 // p25.1	down
11731490_a_at	NUP43	348995	chr6:150045456-150067678 (-) // 68.99 // q25.1	down
11722554_at	NUP88	4927	chr17:5287498-5323355 (-) // 97.75 // p13.2	down
11734501_a_at	NUP98	4928	chr11:3696240-3818892 (-) // 97.51 // p15.4	down
11740934_a_at	NUSAP1	51203	chr15:41624925-41673247 (+) // 97.07 // q15.1	down
11727986_at	ODZ3	55714	chr4:183245096-183724177 (+) // 97.98 // q35.1	down
11736300_a_at	ORMDL1	94101	chr2:190634993-190649075 (-) // 98.13 // q32.2	down
11719881_s_at	OSTM1	28962	chr6:108362614-108395966 (-) // 86.68 // q21	down

11741895_a_at	OTUD4	54726	chr4:146080288-146100832 (-) // 100.0 // q31.21	down
11745194_a_at	OXCT1	5019	chr5:41730168-41870561 (-) // 98.96 // p13.1	down
11716173_a_at	P4HB	5034	chr17:79801133-79818544 (-) // 86.61 // q25.3	down
11722146_s_at	PAIP2B	400961	chr2:71409867-71454237 (-) // 87.87 // p13.3	down
11727545_at	PANK3	79646	chr5:167982377-168006610 (-) // 88.16 // q34	down
11757794_s_at	PAPD5	64282	chr16:50265271-50265735 (+) // 95.26 // q12.1	down
11721318_at	PARM1	25849	chr4:75858287-75975323 (+) // 91.65 // q13.3	down
11726247_s_at	PARP1	142	chr1:226548392-226595827 (-) // 97.66 // q42.12	down
11723950_a_at	PBK	55872	chr8:27667137-27695612 (-) // 97.47 // p21.1	down
11742072_a_at	PBX3	5090	chr9:128510477-128729653 (+) // 98.62 // q33.3	down
11727198_a_at	PCDHA1 /// PCDHA10 /// PCDHA11 /// PCDHA12 /// PCDHA13 /// PCDHA2 /// PCDHA3 /// PCDHA4 /// PCDHA5 /// PCDHA6 /// PCDHA7 /// PCDHA8 /// PCDHA9 /// PCDHAC1 /// PCDHAC2	56134 /// 56135 /// 56136 /// 56137 /// 56138 /// 56139 /// 56140 /// 56141 /// 56142 /// 56143 /// 56144 /// 56145 /// 56146 /// 56147 /// 9752	chr5:140247649-140391929 (+) // 93.16 // q31.3	down
11724553_at	PCGF5	84333	chr10:92979933-93044086 (+) // 92.97 // q23.32	down
11754738_a_at	PDE2A	5138	chr11:72287185-72369165 (-) // 94.61 // q13.4	down
11730906_at	PDS5B	23047	chr13:33160563-33352157 (+) // 99.27 // q13.1	down
11756577_a_at	PEA15	8682	chr1:160175169-160184687 (+) // 93.39 // q23.2	down
11720526_s_at	PEBP1	5037	chr12:118573662-118583389 (+) // 93.96 // q24.23	down
11758315_s_at	PER2	8864	chr2:239152686-239153368 (-) // 99.85 // q37.3	down
11733124_a_at	PEX19	5824	chr1:160246598-160254939 (-) // 89.07 // q23.2	down
11756171_a_at	PFKFB2	5208	chr1:207226384-207250135 (+) // 75.79 // q32.2	down
11739711_a_at	PHACTR2	9749	chr6:143999101-144152321 (+) // 92.55 // q24.2	down

11753313_a_at	PHB2	11331	chr12:7074754-7079831 (-) // 99.41 // p13.31	down
11721972_at	PHF16	9767	chrX:46771867-46920641 (+) // 95.25 // p11.23	down
11728203_a_at	PHGDH	26227	chr1:120254418-120286838 (+) // 95.23 // p12	down
11727180_a_at	PHTF1	10745	chr1:114239828-114302165 (-) // 98.5 // p13.2	down
11754722_s_at	PI4K2B	55300	chr4:25235654-25280713 (+) // 99.91 // p15.2	down
11725842_a_at	PIF1	80119	chr15:65107835-65117867 (-) // 97.57 // q22.31	down
11737862_at	PIGF	5281	chr2:46808423-46844225 (-) // 85.47 // p21	down
11721856_a_at	PIGK	10026	chr1:77554665-77685132 (-) // 85.66 // p31.1	down
11724972_a_at	PIGN	23556	chr18:59711049-59854289 (-) // 94.21 // q21.33	down
11758657_s_at	PKIG	11142	chr20:43247223-43247675 (+) // 98.38 // q13.12	down
11726131_a_at	PKP2	5318	chr12:6586807-6591357 (+) // 90.72 // p13.31 /// chr12:32943678-33049780 (-) // 97.58 // p11.21	down
11730106_a_at	PLCB1	23236	chr20:8112911-8865551 (+) // 98.9 // p12.3	down
11727407_at	PLEKHA3	65977	chr2:179344911-179370090 (+) // 96.88 // q31.2 /// chr19:42026593-42028737 (-) // 63.49 // q13.2	down
11759078_at	PLRG1	5356	chr4:155456156-155471587 (-) // 77.81 // q31.3	down
11726470_s_at	PLS1	5357	chr3:142342227-142432506 (+) // 97.43 // q23	down
11757503_s_at	PNN	5411	chr14:39651574-39652143 (+) // 92.21 // q21.1	down
11716959_s_at	POMGNT1	55624	chr1:46654352-46664111 (-) // 96.51 // p34.1	down
11724413_s_at	PPARA	5465	chr22:46546730-46641621 (+) // 92.86 // q13.31	down
11753001_a_at	PPARG	5468	chr3:12421204-12458653 (+) // 95.66 // p25.2	down
11725616_at	PPP1CB	5500	chr2:28974625-29025806 (+) // 94.87 // p23.2	down
11716069_at	PPPDE1	51029	chr1:244816055-244872335 (+) // 97.71 // q44	down
11725778_a_at	PRDM2	7799	chr1:14031108-14151574 (+) // 96.38 // p36.21	down
11716703_a_at	PRKACB	5567	chr1:84543715-84704692 (+) // 93.04 // p31.1	down
11760103_at	PRKAR2A	5576	chr3:48784018-48885261 (-) // 79.43 // p21.31	down
11754557_s_at	PRKCA	5578	chr17:64804440-64806861 (+) //	down

			85.99 // q24.2	
11735731_a_at	PRKRIR	5612	chr10:54170804-54174413 (+) // 90.94 // q21.1 /// chr3:86061647-86065196 (+) // 90.77 // p12.1 /// chr11:76060999-76091986 (-) // 95.56 // q13.5 /// chr12:43962344-43965950 (-) // 92.1 // q12 /// chr8:79671455-79674772 (-) // 95.03 // q21.12	down
11764058_at	PRNP	5621	chr20:4681519-4682236 (-) // 93.51 // p13	down
11737783_a_at	PROSC	11212	chr8:37620100-37637282 (+) // 88.64 // p11.23	down
11743070_at	PRPF38A	84950	chr1:52870218-52884784 (+) // 98.6 // p32.3	down
11719381_a_at	PSENEN	55851	chr19:36235992-36238417 (+) // 92.5 // q13.12	down
11742933_a_at	PSIP1	11168	chr9:15464063-15511017 (-) // 98.69 // p22.3	down
11732550_at	PTGER2	5732	chr14:52781015-52795976 (+) // 98.65 // q22.1	down
11717341_at	PTGFRN	5738	chr1:117452572-117532975 (+) // 96.47 // p13.1	down
11759479_s_at	PTP4A2	8073	chr1:32372023-32403955 (-) // 88.6 // p35.2	down
11729206_at	PTPN11	5781	chr12:112856535-112947717 (+) // 91.45 // q24.13	down
11749088_s_at	PTPRK	5796	chr6:128290121-128841621 (-) // 99.53 // q22.33	down
11758970_a_at	PVRL3	25945	chr3:110790600-110854801 (+) // 91.74 // q13.13	down
11739313_a_at	PWP1	11137	chr12:108079559-108106257 (+) // 97.13 // q23.3	down
11723801_s_at	PYROXD1	79912	chr12:21590537-21624342 (+) // 89.5 // p12.1 /// chr11:106694628-106698744 (-) // 84.0 // q22.3	down
11718217_at	RAB22A	57403	chr20:56884751-56942563 (+) // 88.45 // q13.32	down
11758481_s_at	RAB3IP	117177	chr12:70213866-70214492 (+) // 30.04 // q15	down
11720798_at	RAB8B	51762	chr15:63481667-63559975 (+) // 98.72 // q22.2	down
11720808_a_at	RABEPK	10244	chr9:127962861-127996437 (+) // 92.46 // q33.3	down
11749520_a_at	RABGAP1	23637	chr9:125795787-125865845 (+) // 95.86 // q33.2	down
11715576_at	RAC1	5879	chr7:6414165-6443601 (+) // 89.77 // p22.1	down
11722870_at	RAD1	5810	chr5:34905367-34916883 (-) // 74.79 // p13.2	down

11728447_at	RAD54B	25788	chr8:95384188-95487343 (-) // 99.42 // q22.1	down
11760837_at	RAET1E	135250	chr6:150204321-150219238 (-) // 58.94 // q25.1	down
11717756_s_at	RALA	5898	chr7:39663081-39747816 (+) // 98.03 // p14.1	down
11748706_a_at	RALY	22913	chr20:32581722-32661841 (+) // 78.01 // q11.22	down
11758403_s_at	RAPH1	65059	chr2:204300665-204301205 (-) // 99.63 // q33.2	down
11760503_a_at	RASSF8	11228	chr12:26208168-26225582 (+) // 97.94 // p12.1	down
11737075_a_at	RBFOX2	23543	chr22:36134783-36424585 (-) // 96.69 // q12.3	down
11717422_s_at	RBM8A	9939	chr1:145507556-145512878 (+) // 83.85 // q21.1 /// chr14:60864424-60867232 (+) // 57.99 // q23.1	down
11729738_x_at	RCCD1	91433	chr15:91498130-91506353 (+) // 51.67 // q26.1	down
11729123_a_at	RCN2	5955	chr15:77223959-77242601 (+) // 98.11 // q24.3	down
11748967_a_at	RDX	5962	chr11:110102281-110167339 (-) // 94.31 // q22.3 /// chr11:4231267-4233528 (-) // 88.67 // p15.4	down
11739729_a_at	RECQL	5965	chr12:21621844-21654603 (-) // 87.18 // p12.1	down
11748631_a_at	REEP5	7905	chr5:112213497-112257936 (-) // 93.86 // q22.2	down
11726839_s_at	RELL1	768211	chr4:37612255-37687999 (-) // 80.18 // p14	down
11718171_a_at	RFWD2	64326	chr1:175913966-176176629 (-) // 97.53 // q25.1	down
11731613_a_at	RHEBL1	121268	chr12:49458327-49463792 (-) // 89.06 // q13.12	down
11716455_at	RHOBTB3	22836	chr5:95066778-95132071 (+) // 89.15 // q15	down
11717417_s_at	RHOU	58480	chr1:228870823-228882416 (+) // 91.44 // q42.13	down
11724691_at	RIBC2	26150	chr22:45809570-45828298 (+) // 96.75 // q13.31	down
11723452_s_at	RIOK2	55781	chr5:96496571-96519005 (-) // 91.59 // q15	down
11725340_a_at	RNASEH1	246243	chr17:16586714-16587543 (+) // 39.07 // p11.2 /// chr17:20805289-20806080 (-) // 39.38 // p11.2 /// chr2:3592676-3606206 (-) // 91.47 // p25.3	down
11732832_a_at	RNASEH2B	79621	chr13:51483891-51544596 (+) // 98.7 // q14.3	down
11747975_x_at	RNF145	153830	chr15:23499339-23501361 (-) //	down

			69.74 // q11.2 /// chr15:20831935-20833957 (-) // 69.67 // q11.2 /// chr5:158585298-158635264 (-) // 97.95 // q33.3	
11725676_a_at	RORA	6095	chr15:60780482-60919729 (-) // 95.82 // q22.2	down
200022_PM_at	RPL18	6141	chr16:76268919-76269536 (-) // 90.16 // q23.1 /// chr19:49118588-49121135 (-) // 96.03 // q13.33	down
11757422_x_at	RPL23	9349	chr17:37006351-37009360 (-) // 70.75 // q12	down
11736721_x_at	RPL32	6161	chr3:12876444-12883081 (-) // 82.08 // p25.2	down
11759114_at	RPL37	6167	chr5:40825363-40835314 (-) // 58.73 // p13.1	down
11736192_at	RRM2B	50484	chr8:103216727-103251346 (-) // 84.81 // q22.3	down
11759693_at	RTKN2	219790	chr10:63997910-64028622 (-) // 93.69 // q21.2	down
11756882_a_at	RTTN	25914	chr18:67671044-67863889 (-) // 97.69 // q22.2	down
11728761_a_at	RUNX1	861	chr21:36160098-36261004 (-) // 94.53 // q22.12	down
11717539_at	RYBP	23429	chr3:72423754-72496264 (-) // 91.39 // p13	down
11724139_s_at	SACS	26278	chr13:23902965-24007867 (-) // 99.21 // q12.12	down
11727336_at	SAMD12	401474	chr8:119201698-119634184 (-) // 94.24 // q24.12	down
11720376_a_at	SAP30L	79685	chr5:153825516-153840613 (+) // 90.35 // q33.2	down
11728906_s_at	SATB2	23314	chr2:200134225-200329831 (-) // 96.67 // q33.1	down
11739478_s_at	SBF2	81846	chr11:9800213-10315754 (-) // 98.64 // p15.4	down
11715897_at	SCARB2	950	chr4:77079889-77135039 (-) // 89.53 // q21.1	down
11756174_s_at	SCD	6319	chr10:102123890-102124583 (+) // 69.04 // q24.31 /// chr17:20692238-20692927 (+) // 66.12 // p11.2	down
11731606_at	SCIN	85477	chr7:12610089-12693227 (+) // 84.64 // p21.3	down
11734521_x_at	SCLT1	132320	chr4:129805151-130014764 (-) // 99.87 // q28.2	down
11717752_s_at	SEC14L1	6397	chr17:75136990-75213183 (+) // 84.8 // q25.2	down
11722354_at	SEC23A	10484	chr14:39501122-39572732 (-) // 98.24 // q21.1	down
11731451_at	SEC62	7095	chr3:169684584-169716161 (+) // 72.06 // q26.2	down

11718862_at	SEC63	11231	chr6:108188961-108279504 (-) // 87.17 // q21	down
11734049_a_at	SEH1L	81929	chr18:12947982-12987533 (+) // 91.31 // p11.21	down
11717204_at	SEL1L	6400	chr14:81937890-82000205 (-) // 97.34 // q31.1	down
11723668_at	SEMA3C	10512	chr7:80371854-80548667 (-) // 96.6 // q21.11	down
11719521_at	SEMA5A	9037	chr5:9035141-9546233 (-) // 95.93 // p15.31	down
11741434_s_at	SEPT10	151011	chr2:110300377-110371783 (-) // 89.56 // q13 /// chr8:57387901-57390834 (-) // 73.13 // q12.1	down
11754612_a_at	SEPT11	55752	chr4:77870918-77959764 (+) // 94.61 // q21.1	down
11736061_a_at	SERPINB5	5268	chr18:61143993-61172317 (+) // 86.19 // q21.33	down
11728413_at	SERPINB8	5271	chr18:61637260-61656600 (+) // 68.88 // q22.1	down
11757442_a_at	SERPINE2	5270	chr2:224839844-224840623 (-) // 90.28 // q36.1	down
11733919_s_at	SERTAD2	9792	chr2:64858754-64881046 (-) // 97.65 // p14	down
11731246_s_at	SERTAD4	56256	chr1:210406143-210416454 (+) // 94.71 // q32.2	down
11756494_a_at	SETD6	79918	chr16:58549433-58553407 (+) // 98.1 // q21	down
11717555_at	SETD7	80854	chr4:140427191-140477577 (-) // 97.2 // q31.1	down
11764283_s_at	SFRS18	25957	chr6:99846538-99847045 (-) // 96.2 // q16.2	down
11756286_a_at	SFXN2	118980	chr10:104474274-104498951 (+) // 81.66 // q24.32	down
11724104_s_at	SGK3	23678	chr8:67624652-67774257 (+) // 83.03 // q13.1	down
11726963_s_at	SGTB	54557	chr5:64961756-65017940 (-) // 70.79 // q12.3	down
11717409_at	SH2B3	10019	chr12:111843751-111889427 (+) // 92.01 // q24.12	down
11718847_at	SH3GLB1	51100	chr1:87170252-87213867 (+) // 75.54 // p22.3	down
11754273_a_at	SH3RF1	57630	chr4:170015362-170192087 (-) // 90.3 // q32.3	down
11747888_a_at	SH3YL1	26751	chr2:218665-264859 (-) // 88.25 // p25.3	down
11728404_at	SHCBP1	79801	chr16:46614466-46655538 (-) // 89.68 // q11.2	down
11763274_a_at	SIPA1L1	26037	chr14:72052997-72207926 (+) // 98.81 // q24.2	down
11722069_a_at	SKA1	220134	chr18:47901391-47920471 (+) // 51.9 // q21.1	down
11722823_a_at	SKP1	6500	chr5:133492081-133512724 (-) //	down

			98.94 // q31.1	
11744052_a_at	SLC11A2	4891	chr12:51379491-51420199 (-) // 97.96 // q13.12	down
11718656_at	SLC16A1	6566	chr1:113454468-113498975 (-) // 99.06 // p13.2	down
11725662_a_at	SLC16A14	151473	chr2:230899695-230933715 (-) // 96.71 // q36.3	down
11723149_a_at	SLC19A2	10560	chr1:169432464-169455241 (-) // 96.39 // q24.2	down
11757680_a_at	SLC1A4	6509	chr2:65249626-65250262 (+) // 96.56 // p14	down
11758222_s_at	SLC20A1	6574	chr2:113420931-113421402 (+) // 93.24 // q13	down
11733119_a_at	SLC35B4	84912	chr7:133973961-134001861 (-) // 91.28 // q33	down
11739254_at	SLC35E1	79939	chr19:16660649-16683193 (-) // 81.5 // p13.11	down
11720075_s_at	SLC35F5	80255	chr2:114471358-114514400 (-) // 96.78 // q14.1	down
11717102_a_at	SLC38A1	81539	chr12:46576837-46663220 (-) // 93.32 // q13.11	down
11739000_a_at	SLC38A2	54407	chr12:46751970-46766650 (-) // 95.78 // q13.11	down
11718461_at	SLC39A11	201266	chr17:70642087-71088853 (-) // 96.13 // q24.3	down
11724252_s_at	SLC39A8	64116	chr4:103182822-103266641 (-) // 95.06 // q24	down
11733492_s_at	SLC44A5	204962	chr1:75667816-76076799 (-) // 97.96 // p31.1	down
11727279_s_at	SLC4A7	9497	chr3:27414213-27498245 (-) // 97.0 // p24.1	down
11722413_a_at	SLC7A1	6541	chr13:30083550-30169847 (-) // 97.86 // q12.3	down
11720989_a_at	SLC7A7	9056	chr14:23242430-23292653 (-) // 97.55 // q11.2	down
11723044_at	SMAD1	4086	chr4:146402910-146480328 (+) // 97.48 // q31.21	down
11720018_a_at	SMAP1	60682	chr6:71377478-71571718 (+) // 96.37 // q13	down
11743341_a_at	SMARCA2	6595	chr9:2021944-2193624 (+) // 92.43 // p24.3	down
11742782_at	SMARCC1	6599	chr3:47626926-47823407 (-) // 95.75 // p21.31	down
11758700_s_at	SMC3	9126	chr10:112362954-112364390 (+) // 96.69 // q25.2 /// chr2:99719049-99719544 (+) // 94.35 // q11.2	down
11754320_a_at	SMC4	10051	chr3:160131589-160152743 (+) // 95.15 // q25.33	down
11724351_a_at	SMC6	79677	chr2:17845079-17935097 (-) // 97.63 // p24.2	down
11717598_s_at	SMPD1	6609	chr11:6411556-6416228 (+) // 95.47 // p15.4	down

11748382_a_at	SMURF2	64750	chr17:62541906-62658079 (-) // 98.4 // q23.3	down
11727830_at	SNAP29	9342	chr22:21213291-21245499 (+) // 84.28 // q11.21	down
11734792_at	SNAPC1	6617	chr14:62229074-62263145 (+) // 81.83 // q23.2	down
11723769_at	SNX24	28966	chr5:122181143-122344902 (+) // 77.77 // q23.2	down
11721960_a_at	SNX30	401548	chr9:115513117-115637267 (+) // 98.25 // q32	down
11761125_at	SNX5	27131	chr20:17927597-17949437 (-) // 68.09 // p11.23	down
11759151_at	SPC24	147841	chr19:11256483-11266484 (-) // 39.8 // p13.2	down
11726158_at	SPIN4	139886	chrX:62567106-62571365 (-) // 62.38 // q11.1	down
11720132_a_at	SPIRE1	56907	chr18:12443294-12658133 (-) // 69.78 // p11.21	down
11734827_at	SPRED1	161742	chr15:38544235-38649449 (+) // 95.01 // q14	down
11743562_a_at	SR140	23350	chr3:142720371-142779567 (+) // 96.27 // q23	down
11747210_a_at	SRI	6717	chr7:87834972-87849351 (-) // 91.28 // q21.12	down
11734789_at	SRR	63826	chr17:2206959-2228553 (+) // 89.66 // p13.3	down
11718777_at	SRRM1	10250	chr1:24969502-24999974 (+) // 92.52 // p36.11	down
11743269_a_at	SRSF1	6426	chr17:56078235-56084707 (-) // 92.16 // q22	down
11759484_s_at	STAG1	10274	chr3:136055077-136471258 (-) // 98.73 // q22.3	down
11720755_at	STAT3	6774	chr17:40465342-40540586 (-) // 91.72 // q21.2	down
11755400_s_at	STK39	27347	chr2:168810530-168969616 (-) // 92.98 // q24.3	down
11719666_a_at	STMN1	3925	chr1:26226530-26232644 (-) // 95.76 // p36.11	down
11724830_at	STS	412	chrX:7137366-7272851 (+) // 87.9 // p22.31	down
11719534_a_at	STX3	6809	chr11:59522478-59570529 (+) // 97.54 // q12.1	down
11758922_at	STXBP6	29091	chr14:25278862-25519095 (-) // 95.83 // q12	down
11720599_s_at	SUB1	10923	chr5:32586219-32604172 (+) // 87.52 // p13.3	down
11721516_at	SUDS3	64426	chr12:118814184-118855840 (+) // 99.96 // q24.23	down
11739486_at	SUZ12	23512	chr17:30264038-30328059 (+) // 96.46 // q11.2	down
11744067_s_at	SYNGR2	9144	chr15:30336642-30338297 (+) // 79.58 // q13.2 /// chr17:76164610-76169013 (+) //	down

			95.72 // q25.3	
11748818_x_at	TADA3	10474	chr3:9821939-9834695 (-) // 100.0 // p25.3	down
11743098_a_at	TARSL2	123283	chr15:102193800-102264807 (-) // 98.98 // q26.3	down
11720224_a_at	TBC1D1	23216	chr4:37892704-38140793 (+) // 98.17 // p14	down
11716928_a_at	TBC1D5	9779	chr3:17198659-17782535 (-) // 89.3 // p24.3	down
11731819_a_at	TBC1D8B	54885	chrX:106045918-106119375 (+) // 97.63 // q22.3	down
11720266_a_at	TBCK	93627	chr4:106967233-107237392 (-) // 99.22 // q24	down
11740213_a_at	TBL1X	6907	chrX:9431105-9687780 (+) // 97.47 // p22.31	down
11742714_a_at	TBRG1	84897	chr11:124492740-124505825 (+) // 77.56 // q24.2	down
11723527_a_at	TCTEX1D2	255758	chr3:196018099-196045159 (-) // 90.57 // q29	down
11718066_a_at	TCTN1	79600	chr12:111051896-111086935 (+) // 95.16 // q24.11	down
11755878_a_at	TESK1	7016	chr9:35605487-35610037 (+) // 94.45 // p13.3	down
11722041_s_at	TEX2	55852	chr17:62224794-62340660 (-) // 97.94 // q23.3	down
11743642_s_at	TFAP2A	7020	chr6:10396915-10412622 (-) // 89.63 // p24.3	down
11716903_a_at	TGOLN2	10618	chr2:85545145-85555407 (-) // 91.76 // p11.2	down
11739446_a_at	TH1L	51497	chr20:57556308-57570168 (+) // 96.86 // q13.32	down
11758842_at	THBS1	7057	chr15:39873133-39891116 (+) // 96.06 // q14	down
11759126_a_at	THRA	7067	chr17:38219062-38249110 (+) // 97.76 // q21.1	down
11725969_a_at	THUMPD1	55623	chr16:20744990-20753199 (-) // 51.05 // p12.3	down
11722786_s_at	TIMP2	7077	chr17:76849058-76921472 (-) // 90.02 // q25.3	down
11726766_x_at	TIPRL	261726	chr1:168148082-168171349 (+) // 96.75 // q24.2	down
11715545_at	TMED10	10972	chr14:75598171-75643349 (-) // 65.49 // q24.3	down
11757430_s_at	TMED5	50999	chr1:93617410-93618009 (-) // 99.17 // p22.1	down
11719539_a_at	TMEM194A	23306	chr12:57449425-57472553 (-) // 98.64 // q13.3	down
11744735_a_at	TMEM200A	114801	chr6:130686518-130764569 (+) // 98.56 // q23.1	down
11716578_at	TMEM43	79188	chr3:14166439-14185179 (+) // 98.67 // p25.1	down
11723540_a_at	TMEM80	283232	chr11:695514-704123 (+) // 96.7 // p15.5	down

11721632_a_at	TMEM97	27346	chr17:26646120-26656088 (+) // 87.71 // q11.2	down
11737296_s_at	TMTC4	84899	chr13:101256090-101327103 (-) // 98.89 // q32.3	down
11721097_at	TMX1	81542	chr14:51706885-51724370 (+) // 91.1 // q22.1	down
11759288_at	TMX4	56255	chr20:7957999-8000521 (-) // 86.09 // p12.3	down
11716063_at	TNC	3371	chr9:117782507-117880818 (-) // 97.71 // q33.1	down
11745020_a_at	TNFRSF12A	51330	chr16:3070359-3072381 (+) // 96.01 // p13.3	down
11736495_a_at	TNRC6B	23112	chr22:40573928-40731809 (+) // 95.57 // q13.1	down
11727782_a_at	TPM4	7171	chr19:16187115-16213813 (+) // 85.24 // p13.12 /// chr3:27674211-27676918 (+) // 79.74 // p24.1	down
11759962_at	TPRKB	51002	chr2:73957387-73964467 (-) // 99.75 // p13.1	down
11720257_a_at	TRAPPC6B	122553	chr14:39617014-39640038 (-) // 67.79 // q21.1	down
11719811_a_at	TRIB3	57761	chr20:361260-378203 (+) // 85.76 // p13	down
11751166_a_at	TRIM5	85363	chr11:5686479-5701462 (-) // 80.7 // p15.4	down
11728915_at	TRIOBP	11078	chr22:38142240-38156253 (+) // 63.08 // q13.1	down
11723437_at	TSGA14	95681	chr7:130034500-130081078 (-) // 96.33 // q32.2	down
11744743_a_at	TSHZ1	10194	chr18:72994818-73001898 (+) // 88.31 // q22.3	down
11722242_at	TSPAN12	23554	chr7:120427373-120498411 (-) // 98.9 // q31.31	down
11735944_x_at	TTC21B	79809	chr2:166730453-166810348 (-) // 92.64 // q24.3	down
11743792_a_at	TTC39C	125488	chr18:21594195-21715932 (+) // 82.15 // q11.2	down
11754362_a_at	TTC7A	57217	chr2:47177559-47303263 (+) // 94.84 // p21	down
11727514_at	TTC9	23508	chr14:71108296-71141812 (+) // 75.88 // q24.2	down
11739206_a_at	TTL	150465	chr2:113239730-113290300 (+) // 84.02 // q13	down
11732219_a_at	TUBE1	51175	chr6:112391860-112408751 (-) // 97.75 // q21	down
11719690_at	TWSG1	57045	chr18:9334764-9402418 (+) // 98.4 // p11.22	down
11723856_at	TXNDC16	57544	chr14:52897307-53019301 (-) // 88.0 // q22.1	down
11752765_s_at	TXNIP	10628	chr1:145438489-145442130 (+) // 99.81 // q21.1	down
11751805_a_at	TYMS	7298	chr18:657742-672997 (+) //	down

			100.0 // p11.32	
11722102_a_at	TYRO3	7301	chr15:41851190-41871536 (+) // 92.99 // q15.1	down
11727817_at	UBASH3B	84959	chr11:122525899-122685185 (+) // 97.6 // q24.1	down
11722028_at	UBE2G1	7326	chr17:4172511-4269973 (-) // 94.74 // p13.2	down
11719284_a_at	UBR7	55148	chr14:93673400-93695560 (+) // 99.27 // q32.12	down
11755633_a_at	UBXN8	7993	chr8:30601705-30624421 (+) // 99.5 // p12	down
11759304_at	UGT8	7368	chr4:115519557-115599744 (+) // 95.72 // q26	down
11735032_a_at	UPRT	139596	chrX:74493895-74524434 (+) // 97.83 // q13.3	down
11745425_a_at	USP25	29761	chr21:17102343-17252377 (+) // 98.02 // q21.1	down
11725800_a_at	USP46	64854	chr4:53456530-53522759 (-) // 84.42 // q12	down
11758383_s_at	USP6NL	9712	chr10:11502510-11503163 (-) // 91.93 // p14	down
11724333_a_at	USP8	9101	chr15:50716578-50793499 (+) // 91.4 // q21.2	down
11724649_at	VANGL1	81839	chr1:116184554-116240842 (+) // 97.62 // p13.1	down
11718191_at	VEZF1	7716	chr17:56048912-56065615 (-) // 96.28 // q22 /// chr3:192871332-192875088 (-) // 71.62 // q29	down
11757631_s_at	VGLL3	389136	chr3:86989786-86990331 (-) // 89.01 // p12.1	down
11715976_a_at	VGLL4	9686	chr3:11597542-11685566 (-) // 97.7 // p25.3	down
11717608_at	VMA21	203547	chrX:150565675-150577836 (+) // 74.51 // q28	down
11747422_a_at	WBSCR22	114049	chr7:73097897-73112323 (+) // 93.18 // q11.23	down
11759217_at	WNT3	7473	chr17:44839871-44896126 (-) // 96.57 // q21.31	down
11732393_a_at	WWC2	80014	chr4:184020445-184241927 (+) // 98.19 // q35.1	down
11728136_at	XK	7504	chrX:37544932-37590348 (+) // 79.34 // p21.1	down
11717920_a_at	YAP1	10413	chr11:101981209-102104149 (+) // 91.79 // q22.1	down
11731397_a_at	YWHAB	7529	chr20:43514185-43537354 (+) // 86.93 // q13.12	down
11726808_at	ZAK	51776	chr2:173940564-174132737 (+) // 91.18 // q31.1	down
11727339_a_at	ZBTB2	57621	chr6:151685172-151712835 (-) // 98.52 // q25.1	down
11725870_at	ZBTB41	360023	chr1:197122742-197169721 (-) // 92.14 // q31.3	down

11730939_at	ZBTB8A	653121	chr1:33004743-33071551 (+) // 73.39 // p35.1	down
11729839_at	ZCCHC2	54877	chr18:60190657-60245812 (+) // 95.24 // q21.33	down
11721528_at	ZDHHC2	51201	chr8:17013408-17080793 (+) // 96.01 // p22	down
11725596_a_at	ZDHHC23	254887	chr3:113666747-113681829 (+) // 95.26 // q13.31	down
11726524_s_at	ZFHX3	463	chr16:72816787-73082274 (-) // 95.7 // q22.2	down
11733145_at	ZFP161	7541	chr18:5289018-5296194 (-) // 98.3 // p11.31	down
11726600_s_at	ZMPSTE24	10269	chr1:40723721-40759856 (+) // 98.16 // p34.2	down
11739975_at	ZNF117	51351	chr7:64432793-64442497 (-) // 67.72 // q11.21	down
11721601_s_at	ZNF238	10472	chr1:244216506-244220778 (+) // 92.03 // q44	down
11739896_at	ZNF280C	55609	chrX:129336672-129402922 (-) // 89.69 // q26.1	down
11762388_at	ZNF280D	54816	chr15:56944687-56999506 (-) // 95.47 // q21.3	down
11755232_s_at	ZNF468	90333	chr6:163881899-163883249 (+) // 62.04 // q26	down
11733207_a_at	ZNF480	147657	chr19:52800420-52826572 (+) // 39.83 // q13.41	down
11758902_at	ZNF641	121274	chr12:48730959-48744554 (-) // 85.12 // q13.11	down
11721524_s_at	ZNF706	51123	chr8:102209271-102217960 (-) // 97.31 // q22.3	down

Supplementary Table 5: the results of the Go analysis between the differently expressed genes

GO analysis (molecular function)			
Gene Set Name	Gene number	Significant probability	Gene name
CHEMOKINE_ACTIVITY	3	9.30E-02	CCL20,CXCL3,IL8
CHEMOKINE_RECEPTOR_BINDING	3	9.82E-02	CCL20,CXCL3,IL8
PROTEIN_KINASE_INHIBITOR_ACTIVITY	2	1.26E-01	CDKN2B,PKIG
KINASE_INHIBITOR_ACTIVITY	2	1.35E-01	CDKN2B,PKIG
G_PROTEIN_COUPLED_RECEPTOR_BINDING	3	1.62E-01	CCL20,CXCL3,IL8
PROTEIN_KINASE_REGULATOR_ACTIVITY	2	2.66E-01	CDKN2B,PKIG
UDP_GLYCOSYLTRANSFERASE_ACTIVITY	2	2.95E-01	MGAT2,GALNT7
CYTOKINE_ACTIVITY	4	3.33E-01	CCL20,CXCL3,IL8,SPRED1

KINASE_REGULATOR_ACTIVITY	2	3.33E-01	CDKN2B,PKIG
PHOSPHOLIPID_BINDING	2	3.43E-01	PIGK,PLEKHA 3
GO analysis (biological process)			
REGULATION_OF_TRANSFORMING_GRO WTH_FACTACTOR_BETA_RECEPTOR	2	3.73E-02	CDKN2B,HIPK 2
TRANSMEMBRANE_RECEPTOR_PROTEIN SERINE_NE_THREONINE_KINASE_SIGNA LI NG_PATHWAY	3	8.54E-02	CDKN2B,HIPK 2,BMPR2
REGULATION_OF_SMALL_GTPASE_MEDI A TED_SI_SIGNAL_TRANSDUCTION	2	9.13E-02	MFN2,CDC42B PA
ORGANELLE_LOCALIZATION	2	1.05E-01	MFN2,SNAP29
CELL_CYCLE_ARREST_GO_0007050	3	1.32E-01	CDKN2B,MFN 2,IL8
NEGATIVE_REGULATION_OF_TRANSFER AS E_ACTACTIVITY	2	1.81E-01	SPRED1,TRIB3
TRANSFORMING_GROWTH_FACTOR_BET A _RECEPTEPTOR_SIGNALING_PATHWAY	2	1.90E-01	CDKN2B,HIPK 2
REGULATION_OF_CELL_ADHESION	2	1.98E-01	IL8,ARHGDIA
NEGATIVE_REGULATION_OF_CELL_CYCL E	3	2.55E-01	CDKN2B,MFN 2,IL8
SMALL_GTPASE_MEDIATED_SIGNAL_TR A NSDUCTUCTION	3	3.16E-01	MFN2,CDC42B PA,ARH GDIA
GO analysis (cellular component)			
VOLTAGE_GATED_POTASSIUM_ CHANNEL_COMPLEPLEX	2	2.79E-01	KCNJ8,KCNQ1
INTRINSIC_TO_ORGANELLE_ME	2	3.94E-01	PIGK,MFN2

Supplementary Table 6: the results of the pathway analysis of the differently expressed genes

pathway analysis result			
Gene Set Name	Gene number	Significant	Gene name
BIOCARTA_AGPCR_PATHWAY	2	3.94E-02	PRKCA,PRKAR2A
BIOCARTA_CBL_PATHWAY	2	3.94E-02	PRKCA,SH3GLB1
BIOCARTA_CDMAC_PATHWAY	2	5.78E-02	PRKCA,PLCB1
BIOCARTA_CK1_PATHWAY	2	6.45E-02	PRKAR2A,PLCB1
BIOCARTA_SPPA_PATHWAY	2	1.01E-01	PRKCA,PLCB1
BIOCARTA_CCR3_PATHWAY	2	1.09E-01	PRKCA,PLCB1
BIOCARTA_NOS1_PATHWAY	2	1.17E-01	PRKCA,PRKAR2A
BIOCARTA_TPO_PATHWAY	2	1.17E-01	PRKCA,STAT3
BIOCARTA_CREB_PATHWAY	2	1.42E-01	PRKCA,PRKAR2A
BIOCARTA_EDG1_PATHWAY	2	1.42E-01	PRKCA,PLCB1

Supplemental Table 7: the Primer sequences used in the study

Gene	Forward Sequence	Reverse Sequence	Size (bp)
GAPDH	TGACTTCAACAGCGACACCCA	CACCCTGTTGCTGTAGCCAAA	121
SCIN	TCTGCGTTCCTGACTGTTC	GACCTCCTTTCTTTGATGTTCC	154
CDKN2C	AGACTGCTACTTAGAGGTGCTA	CAGGTTCCCTTCATTATCC	153
CDKN2B	CTGGACCTGGTGGCTACG	ACATTGGAGTGAACGCATCG	114
DIAPH1	CAGTTGGGTGCAAACATTTGG	TCCGGCTATCGTAACTCCCAG	107
MCM4	TATCTAAGGGCAAAACAC	CAGTCACTGTCAGGAAAT	141
STAT3	GACATGGAGTTGACCTCGGAGTG	GGTGGCAGAATGCAGGTAGGC	103
EGFR	ATGAGATGGAGGAAGACGG	CGGCAGGATGTGGAGAT	173
RAC1	ATGTCCGTGCAAAGTGGTATC	CTCGGATCGCTTCGTCAAACA	249
DHFR	CTGTCTTAGATTGGGGAACCC	ATGAGAACCTGCTCGCTGA	158
CDK2	CTGGACACTGAGACTGAGG	GAGGACCCGATGAGAATGG	264
COL4A1	GGACTACCTGGAACAAAAGGG	GCCAAGTATCTCACCTGGATCA	240
PRKCA	TGGACTTATCCATCAAGGGATGA	AGTGTGATCCATTCCGCAGAG	100
CDK4	AGTGGTGGAACAGTCAAG	AGCCCAATCAGGTCAAAG	248
CCNB1	CTAAGATTGGAGAGGTTGATGTC	GGTAATGTTGTAGAGTTGGTGTC	177
ARHGAP5	ACTGCACTGGTACAAATGTTGG	TCACAGTCTGCACAAGTTTTTCA	133
ITGB1	CCTACTTCTGCACGATGTGATG	CCTTTGCTACGGTTGGTTACATT	128
PLCB1	GCCTCGCCAGAAAAGGACAA	CATCTCAACAGAGGAGTTTCCAG	197
